# Alterations in Gene Expression in Mutant Amyloid Precursor Protein Transgenic Mice Lacking Niemann-Pick Type C1 Protein

**DOI:** 10.1371/journal.pone.0054605

**Published:** 2013-01-29

**Authors:** Mahua Maulik, Gopal Thinakaran, Satyabrata Kar

**Affiliations:** 1 Centre for Prions and Protein Folding Diseases, University of Alberta, Edmonton, Alberta, Canada; 2 Centre for Neuroscience, University of Alberta, Edmonton, Alberta, Canada; 3 Departments of Neurobiology, Neurology and Pathology, The University of Chicago, Chicago, Illinois, United States of America; 4 Departments of Medicine (Neurology) and Psychiatry, University of Alberta, Edmonton, Alberta, Canada; Nathan Kline Institute and New York University School of Medicine, United States of America

## Abstract

Niemann-Pick type C (NPC) disease, a rare autosomal recessive disorder caused mostly by mutation in *NPC1* gene, is pathologically characterized by the accumulation of free cholesterol in brain and other tissues. This is accompanied by gliosis and loss of neurons in selected brain regions, including the cerebellum. Recent studies have shown that NPC disease exhibits intriguing parallels with Alzheimer’s disease, including the presence of neurofibrillary tangles and increased levels of amyloid precursor protein (APP)-derived β-amyloid (Aβ) peptides in vulnerable brain neurons. To evaluate the role of Aβ in NPC disease, we determined the gene expression profile in selected brain regions of our recently developed bigenic ANPC mice, generated by crossing APP transgenic (Tg) mice with heterozygous Npc1-deficient mice. The ANPC mice exhibited exacerbated neuronal and glial pathology compared to other genotypes [i.e., APP-Tg, double heterozygous (Dhet), Npc1-null and wild-type mice]. Analysis of expression profiles of 86 selected genes using real-time RT-PCR arrays showed a wide-spectrum of alterations in the four genotypes compared to wild-type controls. The changes observed in APP-Tg and Dhet mice are limited to only few genes involved mostly in the regulation of cholesterol metabolism, whereas Npc1-null and ANPC mice showed alterations in the expression profiles of a number of genes regulating cholesterol homeostasis, APP metabolism, vesicular trafficking and cell death mechanism in both hippocampus and cerebellum compared to wild-type mice. Intriguingly, ANPC and Npc1-null mice, with some exceptions, exhibited similar changes, although more genes were differentially expressed in the affected cerebellum than the relatively spared hippocampus. The altered gene profiles were found to match with the corresponding protein levels. These results suggest that lack of Npc1 protein can alter the expression profile of selected transcripts as well as proteins, and APP overexpression influences cerebral pathology by enhancing changes triggered by Npc1 deficiency in the bigenic line.

## Introduction

Niemann-Pick type C (NPC) disease is an autosomal recessive neurovisceral disorder caused predominantly by mutations in the *NPC1* gene and less frequently in the *NPC2* gene. The *NPC1* gene encodes for a 1278 amino acid polytopic membrane protein harboring a sterol sensing domain, whereas *NPC2* gene encodes for a soluble cholesterol binding protein. The loss of function of either protein leads to intracellular accumulation of unesterified cholesterol and glycosphingolipids in many tissues, including the brain. These defects in cholesterol sequestration trigger widespread neurological deficits such as ataxia, dystonia, seizures and dementia leading to premature death [1], [2], [3]. In addition to cholesterol accumulation, NPC disease is neuropathologically characterized by the presence of tau-positive neurofibrillary tangles, gliosis, demyelination and loss of neurons in selected brain regions [2], [4], [5]. Moreover, NPC patients carrying Apolipoprotein E (APOE) ε4 alleles develop extracellular cerebral deposition of β-amyloid (Aβ) peptides [6], a characteristic pathological feature of Alzheimer’s disease (AD), the most common type of senile dementia affecting the elderly [7], [8], [9], [10]. Some recent studies have also reported increased levels of Aβ-related peptides in vulnerable neurons as well as in the cerebrospinal fluid of NPC patients [11], [12]. Although overall increase in the level or intracellular accumulation of cholesterol is known to trigger generation of Aβ peptides by proteolytic processing of amyloid precursor protein (APP), the functional significance of these peptides in NPC pathology remains unclear [13], [14], [15], [16].

Earlier studies have shown that BALB/c-*Npc1^nih^* mice, which do not express Npc1 protein (Npc1-null) due to a spontaneous mutation in the *Npc1* gene, can recapitulate most of the pathological features associated with human NPC disease, with the exception of neurofibrillary tangles [17], [18], [19], [20]. These Npc1-null mice are usually asymptomatic at birth but gradually develop tremor and ataxia, and die prematurely at ∼3 months of age. At the cellular level, these mice exhibit intracellular accumulation of cholesterol, activation of microglia and astrocytes as well as loss of myelin sheath throughout the central nervous system. Progressive loss of neurons is also evident in selected brain regions including cerebellum, whereas the hippocampus is relatively spared [20], [21], [22]. These mice exhibit increased levels of intracellular Aβ-related peptides in distinct brain regions [11], but the significance of Aβ in the development and/or progression of NPC disease pathology remain unclear. To evaluate the potential role of Aβ peptides in pathological abnormalities related to NPC disease, we have recently developed a new line of bigenic ANPC mice by crossing heterozygous Npc1-deficient mice with mutant human APP transgenic (APP-Tg) mice which exhibit extracellular Aβ deposits and spatial learning deficits but no overt loss of neurons in any brain region. These bigenic mice clearly show that APP overexpression can increase the rate of mortality and exacerbate behavioral as well as neuropathological abnormalities associated with Npc1-null phenotype [23]. Thus, these mice provide a suitable model system to evaluate how expression of human APP in the absence of functional Npc1 protein can influence pathological abnormalities related to AD and NPC disease. Here, we used a gene expression profiling approach to probe the molecular basis underlying the accelerated development of pathological abnormalities in ANPC mice. Specifically, we focused on the expression of 86 selected genes that are involved in APP and Aβ metabolism, cholesterol homeostasis, intracellular vesicular trafficking and cell death mechanisms in the affected cerebellar and relatively spared hippocampal regions of ANPC, Npc1-null, APP-Tg and double heterozygous (Dhet) mice compared to wild-type (WT) controls. The alterations in gene expression profiles were validated using Western blotting. Our results clearly show that Npc1-null and ANPC mice exhibit marked alterations in the expression profiles of a number of genes in both hippocampus and cerebellum, while the changes in APP-Tg and Dhet mice are limited to only few genes, mostly in the hippocampus, compared to WT mice. Furthermore, the alterations in Npc1-null and ANPC mice, consistent with the severity of disease pathology, are more pronounced in the affected cerebellar region than the hippocampus.

## Materials and Methods

### Materials

4–12% NuPAGE Bis-Tris gels, Prolong Gold Antifade and Alexa Fluor-488/594 conjugated secondary antisera were purchased from Life Technologies Corp. (Burlington, ON, Canada). The bicinchoninic acid protein assay kit and enhanced chemiluminescence (ECL) kit were obtained from Thermo Fisher Scientific (Montreal, QC, Canada). DNA isolation kit, RNeasy lipid tissue mini kit, SABiosciences’ real-time RT-PCR First Strand Kit, real-time RT-PCR SYBR Green/Fluorescein qPCR master mix and 96-well customized real-time RT-PCR Array were all from Qiagen Inc. (Mississauga, ON, Canada). Polyclonal anti-Apoe antibody was a gift from Dr. J.E. Vance (University of Alberta, AB, Canada), polyclonal anti-Abca1 antibody was provided by Dr. S. Sipione (University of Alberta, AB, Canada), polyclonal anti-Igf2r antibody was a gift from Dr. C. Scott (Kolling Institute of Medical Research, New South Wales, Australia) and polyclonal anti-Npc2 antibody was a gift from Dr. P. Lobel (University of Medicine and Dentistry of New Jersey, NJ, USA). Polyclonal anti-Aplp1 antibody was generated in our laboratory. Monoclonal anti-tau (clone Tau 5) and anti-NeuN antisera were from EMD Millipore Corp. (Billerica, MA), whereas polyclonal anti-β-glucoronidase was from Novus Biologicals (Oakville, ON, Canada). Monoclonal anti-neprilysin and polyclonal anti-Ide antisera were from Abcam (Cambridge, MA). Monoclonal anti-Calbindin-D28K, anti-β-actin and anti-Gapdh antisera as well as filipin were from Sigma-Aldrich (Oakville, ON, Canada), whereas monoclonal anti-Gsk3β was from BD Transduction Laboratories™ (Mississauga, ON, Canada). Polyclonal anti-cathepsin B, anti-cathepsin D antisera and all horseradish peroxidase-conjugated secondary antibodies were from Santa Cruz Biotechnology, Inc (Paso Robles, CA). All other chemicals were from Sigma-Aldrich or Thermo Fisher Scientific.

### Generation of Transgenic Mice

Mutant human APP_KM670/671NL+V717F_ Tg mice (APP-Tg) maintained on a C3H/C57BL6 background [24] and heterozygous Npc1-null mice [19] maintained on a Balb/cNctr-*Npc1^m1N^*/J background were from our breeding colony. These mutant mice were first crossed to produce *APP^+/0^Npc1^+/−^* and *APP^0/0^Npc1^+/−^* off-springs (0/0 represents the absence of human *APP* transgene, whereas 0/+ represents hemizygous for human *APP* transgene), which were subsequently crossed to generate the following five lines of mice: bigenic *APP^+/0^Npc1^−/−^* (ANPC), *APP^+/0^Npc1^+/+^* (APP-Tg), *APP^0/0^Npc1^−/−^* (Npc1-null), *APP^+/0^Npc1^+/−^* (double heterozygous: Dhet) and *APP^0/0^Npc1^+/+^* (wild-type: WT). In this study we used 7-week-old mice from different genotypes because of two reasons i) the mortality rate of ANPC mice increases drastically from ∼8 weeks onwards and ii) distinct behavioral and neuropathological abnormalities are present in ANPC mice compared to other genotypes [19]. All animals were bred and housed with access to food and water *ad libitum*. This study was carried out in strict accordance with the recommendations of the Canadian Council on Animal Care guidelines. The protocol was approved by the Health Sciences Animal Care and Use Committee of the University of Alberta (protocol # 405/07/12/D). All experiments were performed under Isoflurane anesthesia and all efforts were made to minimize the suffering of animals. Different lines of transgenic mice were genotyped by PCR analysis of tail DNA as described earlier [19], [24].

### Histology and Immunohistochemistry

WT, APP-Tg, Dhet, Npc1-null and ANPC mice (n = 4–5 per genotype) of 7-weeks of age were transcardially perfused and fixed in 4% paraformaldehyde. Brains were sectioned on a cryostat (20 µm) and then processed as described earlier [21]. To determine cholesterol accumulation, hippocampal and cerebellar sections from all five genotypes were incubated with 25 µg/ml of filipin in phosphate-buffered saline for 30 min in the dark under agitation [21]. For immunohistochemistry, brain sections were incubated overnight at 4°C with anti-Calbindin-D-28 k (1∶7000) or anti-NeuN (1∶25,000) antibodies. Subsequently, sections were processed with Alexa Fluor 488/594 conjugated secondary antibodies (1∶1000) for immunofluo-rescence methods and examined using a Zeiss Axioskop-2 microscope (Carl Zeiss Canada Ltd.).

### RNA Extraction for PCR Array

Total RNA was isolated from hippocampal and cerebellar tissues of 7-week old ANPC, Dhet, Npc1-null, APP-Tg and WT mice (4 animals per genotype for each brain region studied) using RNeasy lipid tissue mini kit following manufacturer’s instructions (Qiagen Inc., Mississauga, ON, Canada) and stored at −80°C. RNA concentrations were determined using a Nanodrop 1000 spectrophotometer (Thermo Fisher Scientific) and 260/230 nm and 260/280 nm absorbance ratio were analyzed to determine RNA purity.

### Real-time RT-PCR Array

At first 1 µg of total RNA was treated with genomic DNA elimination buffer at 42°C for 5 mins to remove possible genomic DNA contamination. Following the elimination step, reverse transcription was carried out using the real-time RT-PCR First Strand Kit in accordance with the manufacturer’s protocol (SuperArray Biosciences Corp., MD). The resulting complementary DNA (cDNA) was diluted and combined with real-time RT-PCR SYBR Green/Fluorescein qPCR master mix and loaded onto a 96-well customized real-time RT-PCR Array designed to profile the expression of 86 genes representative of biological pathways involved in cholesterol and APP metabolism, intracellular trafficking and cell death. All real-time PCR reactions were performed in a final volume of 25 µl using a MyiQ™ Real-Time PCR Detection System (Bio-Rad Laboratories, Inc., Canada) using a two-step cycling program: 10 min at 95°C (one cycle), 15 s at 95°C, followed by 1 min at 60°C (40 cycles). Data collection was performed during the annealing step (58°C) of each cycle and data were PCR-baseline subtracted and curve fitted. Threshold cycle (Ct) values were calculated using the instrument’s MyiQ optical software (Bio-Rad Laboratories, Inc.).

### PCR Data Normalization and Analysis

The data were analyzed using the SABiosciences’ PCR Array Data analysis software based on the comparative Ct method and expressed as relative fold differences in APP-Tg, Dhet, Npc1-null or ANPC mice compared to WT mice. All Ct values ≥35 were considered a negative call. Quality control tests for PCR reproducibility, reverse transcription efficiency, and level of genomic DNA contamination were included in each plate and monitored as per the supplier’s instructions. The expression level of three housekeeping genes included in the PCR array: *Hprt1*, *Gapdh* and *Actb* were used for normalization. The ΔCt for each gene in each plate was first calculated by subtracting the Ct value of the gene of interest from the average Ct value of the three housekeeping genes. Then, the average ΔCt value of each gene was calculated across the four replicate arrays for each animal group within the same brain region and ΔΔCt values were obtained by subtracting the ΔCt values of WT group from the respective ΔCt values of APP-Tg, Dhet, Npc1-null or ANPC mice. The fold change for each gene from WT to APP-Tg, Dhet, Npc1-null or ANPC mice was calculated as 2∧(-ΔΔCt). Finally the “fold-change” for each gene was converted to “fold-regulation” as follows. For fold-change values greater than 1, which indicated a positive or an up-regulation, the fold-regulation was equal to the fold-change. For fold-change values less than 1 indicating a negative or down-regulation, the fold-regulation was calculated as the negative inverse of the fold-change. *P*-values were calculated using Student’s t-test. A fold difference of ≥1.2 with a *p*-value <0.05 was considered as significant differential gene expression.

The gene expression profiles that were selectively altered in APP-Tg mice compared to WT mice include up-regulation of *Acat2*, *Sqle*, *Fdps*, *Fdft1* and *Dhcr24* in the hippocampus but not in the cerebellum. The genes that were down-regulated in APP-Tg mice include *Cyp46a1* in the hippocampus and *Park2* in the cerebellum. The Dhet mice, on the other hand, showed significant (*p*<0.05) up-regulation of *Fdft1*, *Dhcr24* and *Sqle* and down-regulation of *Ctsb*, *Srebf1* and *Cyp46a1* in the hippocampus, whereas no alteration of any gene was evident in the cerebellum. The majority of the differentially expressed genes in our data set showed 1.2–2 fold changes, whereas only few genes such as *Ctsd*, *Gusb*, *A2m*, *Npc2*, *Apoe*, *Plau* and *Bid* displayed more than 2 fold changes compared to WT mice. These changes were found in Npc1-null or ANPC mice. The majority of the 86 transcripts evaluated in our study including *Aplp1*, *Ide*, *Igf2r* and *Gsk3β*, however, did not exhibit any alterations either in the hippocampus or cerebellum among fives lines of mice (Figs. 8 and 9).

### Western blotting

Hippocampal and cerebellar regions of 7 week old ANPC mice and their age-matched siblings (n = 4–6 animals/genotype) were processed for Western blotting as described earlier [21]. In brief, tissues were homogenized in RIPA lysis buffer and equal amount of proteins were separated on 4–12% NuPAGE Bis-Tris gels. The proteins were transferred to PVDF membranes and incubated overnight at 4°C with anti-Aplp1 (1∶1000), anti-Ide (1∶500), anti-Igf2r (1∶5000), anti-Gsk3β (1∶5000), anti-neprilysin (1∶500), anti-Npc2 (1∶500), anti-Abca1 (1∶500), anti-Apoe (1∶5000), anti-tau (1∶2000), anti-cathepsin B (1∶500), anti-cathepsin D (1∶500) or anti-β-glucoronidase (1∶1000) antisera. Membranes were then exposed to respective secondary antibodies and visualized using an ECL detection kit. Blots were reprobed with anti-β-actin (1∶5000) and/or anti-GAPDH (1∶1000) and quantified using a MCID image analyzer (Imaging Research, Inc.) as described earlier [21]. The data are expressed as mean ± S.E.M and statistically analyzed using one-way ANOVA followed by Newman-Keuls post-hoc analysis with significance set at *p*<0.05. All statistical analyses were performed using GraphPad Prism (GraphPad software, Inc., CA, USA).

## Results

### Real-time RT-PCR Array Analysis of Gene Expression

**Figure 1:**
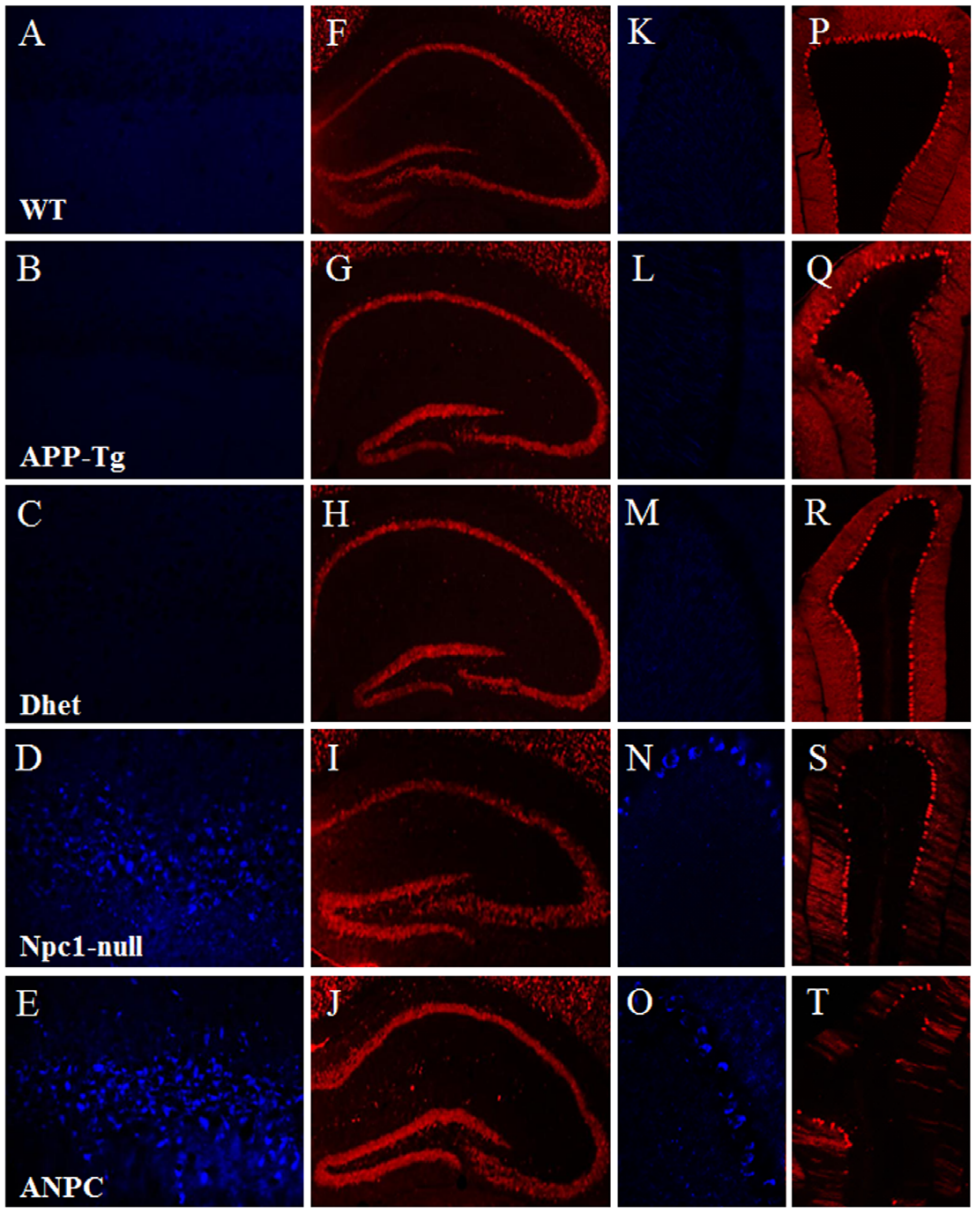
Cholesterol accumulation and neurodegeneration in Npc1-null and ANPC mice compared to other genotypes. **A–E,** Photomicrographs showing filipin staining of unesterified cholesterol in the hippocampus of WT, APP-Tg, Dhet, Npc1-null and ANPC mice. Cholesterol accumulation is evident only in the hippocampal neurons of Npc1-null (D) and ANPC (E) mice but not in WT (A), APP-Tg (B) or Dhet (C) littermates. **F–J,** Photomicrographs showing NeuN labeled hippocampal sections from mice of different genotypes. No apparent loss of neurons was evident in the hippocampus of Npc1-null (I) or ANPC (J) mice compared to WT (F), APP-Tg (G) and Dhet (H) littermates. K–O, Photomicrographs showing filipin staining in the cerebellum of WT, APP-Tg, Dhet, Npc1-null and ANPC mice. Accumulation of cholesterol is evident only in the cerebellar neurons of Npc1-null (N) and ANPC (O) mice but not in WT (K), APP-Tg (L) or Dhet (M) mice. P–T, Photomicrographs showing calbindin-positive cerebellar Purkinje cell layer (Pcl) in mice from different genotypes. Note the relative loss of Purkinje cells in Npc1-null (S) and ANPC (T) mice compared to WT (P), APP-Tg (Q) and Dhet (R) littermates. All photomicrographs of each column are of same magnification. CA1–CA3, Cornu Ammonis1–3 subfields of the Ammon’s horn; DG, dentate gyrus; Pcl, Purkinje cell layer.

The mutant APP-Tg mice used in this study exhibit extracellular Aβ deposits and cognitive behavioral deficits, but no overt loss of neurons or neurofibrillary tangles in any brain region by 3 months of age [24], [25]. The Npc1-null mice, on the other hand, exhibit intracellular cholesterol accumulation and loss of cerebellar Purkinje cells, but lack extracellular Aβ deposits. These mice survive for 12–16 weeks after birth and do not exhibit any significant loss of neurons in the hippocampal region [18], [19], [20]. The bigenic ANPC mice, in our colony, survived for ∼11 weeks after birth, but their mortality rate increased considerably from the 8^th^ week onwards. These mice exhibited significant cognitive and motor deficits by 7-weeks of age compared to other littermates [23]. At the cellular level, ANPC mice accumulate filipin-labeled unesterified cholesterol in most of the neurons of the hippocampus and cerebellum as observed in Npc1-null mice. No cholesterol accumulation was apparent in the brains of WT, APP-Tg or Dhet littermates (Fig. 1). We also observed the presence of degenerating neurons (i.e., Purkinje cells) in the cerebellum, but not in the hippocampus, of ANPC and Npc1-null mouse brains (Fig. 1). Age-matched WT, APP-Tg or Dhet mice did not show cell loss either in the hippocampus or cerebellum. Accompanying these changes, ANPC mice showed profound activation of astrocytes and microglia in a manner which exceeded the level and intensity of staining noted in other genotypes [23].

**Figure 2:**
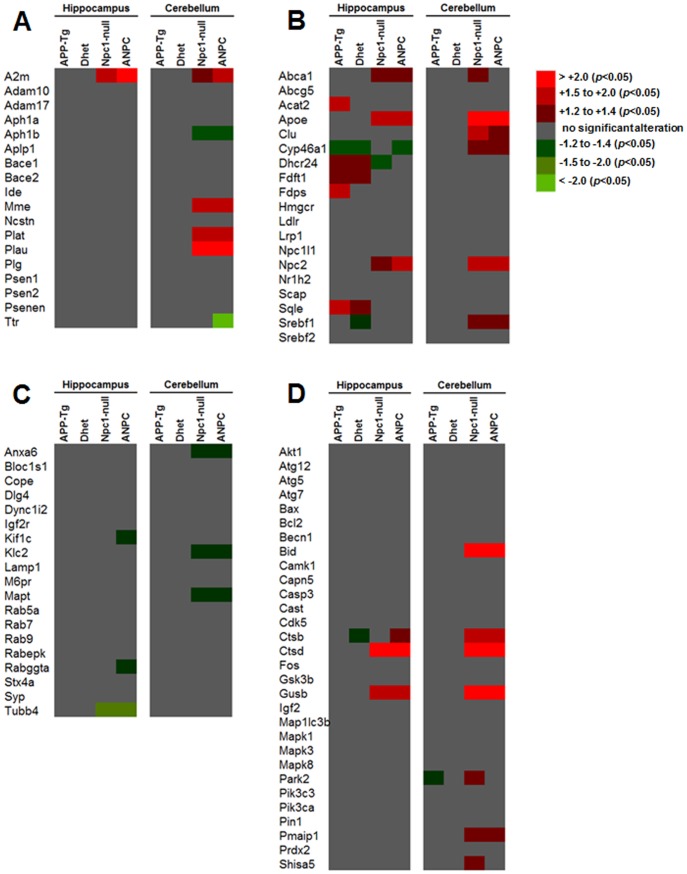
Heat-map diagram showing gene expression profiles in five lines of mice. The figure represents data obtained using customized real-time RT-PCR array of 86 selected genes involved in brain APP (A) and cholesterol (B) metabolisms, intracellular vesicular trafficking (C) and cell death pathways (D) in the hippocampus and cerebellum of 7-week-old APP-Tg, Dhet, Npc1-null and ANPC mice compared with WT littermates. Each row represents a single gene and each column represents a mouse genotype combination. Expression levels are colored red for significant up-regulations, green for significant down-regulations and grey for no alteration compared with WT mice. As shown, the major changes in gene expression occurred in the hippocampus and cerebellum of Npc1-null and ANPC mice while changes in APP-Tg and Dhet mice are limited to only a few genes mainly in the hippocampus. A fold difference of ≥1.2 with a *p*<0.05 was considered to be a significant dysregulation.

**Figure 3:**
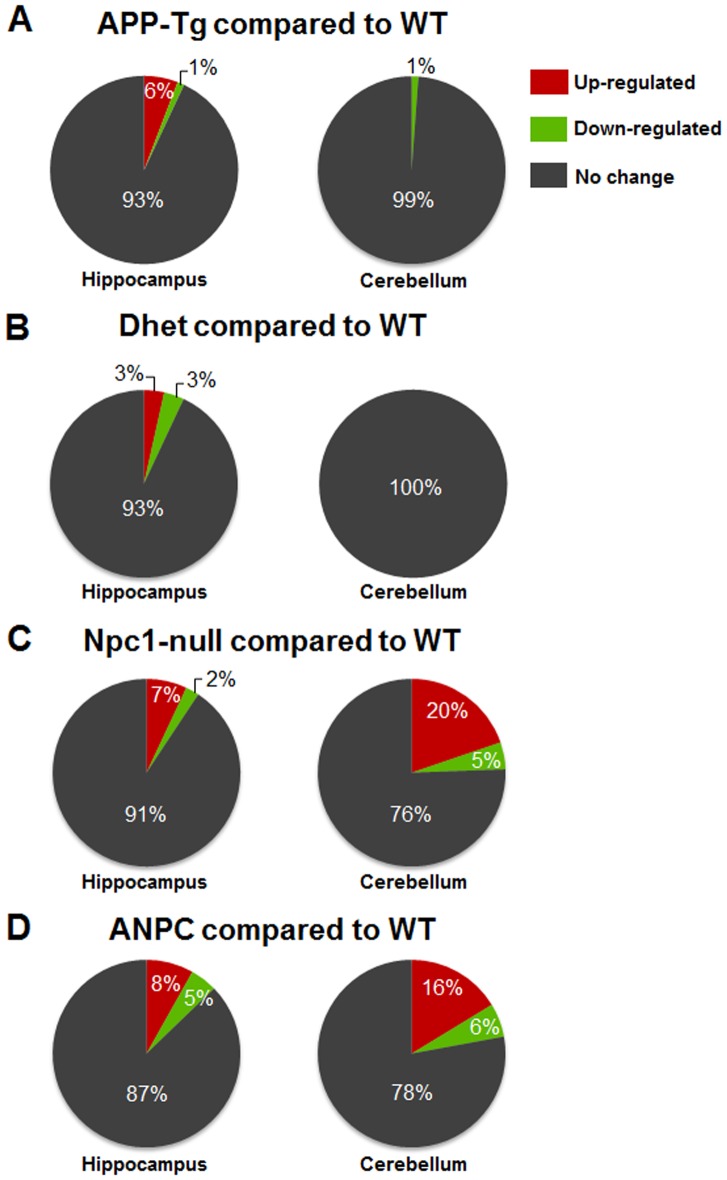
Differentially expressed genes in different mice lines compared to WT mice. Pie-charts showing percentage of up- and down-regulated genes in the hippocampus and cerebellum of 7-week-old APP-Tg (A), Dhet (B), Npc1-null (C) and ANPC (D) mice each compared to WT littermates. Gene expression levels are colored red for significant up-regulation, green for significant down-regulations and grey for no alteration compared to WT mice. As evident from the pie-charts, several genes are differentially expressed in the hippocampus and cerebellum of Npc1-null and ANPC mice, whereas the changes in APP-Tg and Dhet mice are limited to only a few genes.

**Figure 4:**
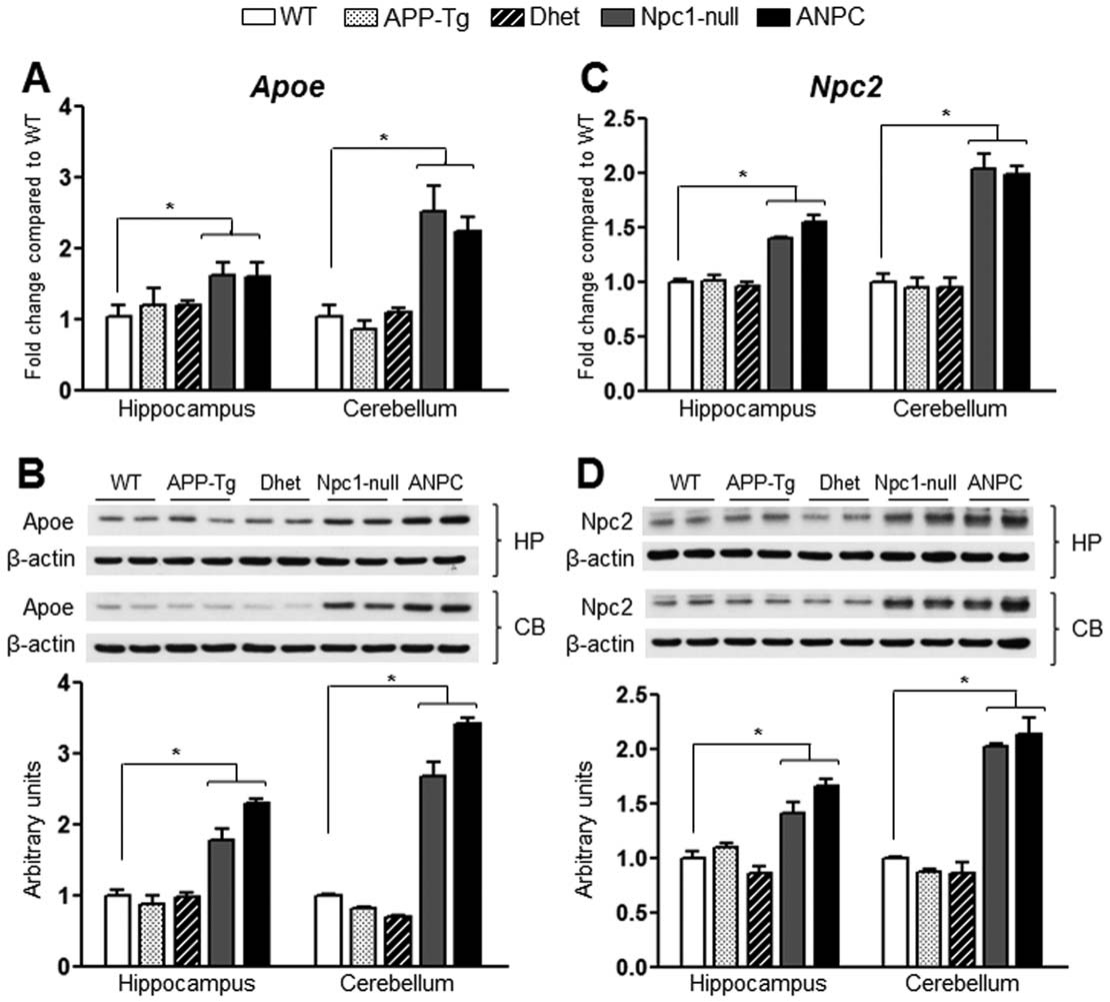
Transcript and protein expression levels of Apoe and Npc2 in the hippocampus and cerebellum of five lines of mice. **A and C,** Histograms showing increased mRNA levels for *Apoe* (A) and *Npc2* (C) in the hippocampus and cerebellum of Npc1-null and ANPC mice compared with WT mice as obtained using customized real-time RT-PCR array. **B and D,** Immunoblots and respective histograms validating increased levels of Apoe (B) and Npc2 (D) in the hippocampus and cerebellum of Npc1-null and ANPC mice compared with age-matched WT mice. The protein levels of Apoe and Npc2 were normalized to the β-actin and the values (n = 4 animals per genotype) are expressed as means ± SEM. *, *p*<0.05.

**Figure 5:**
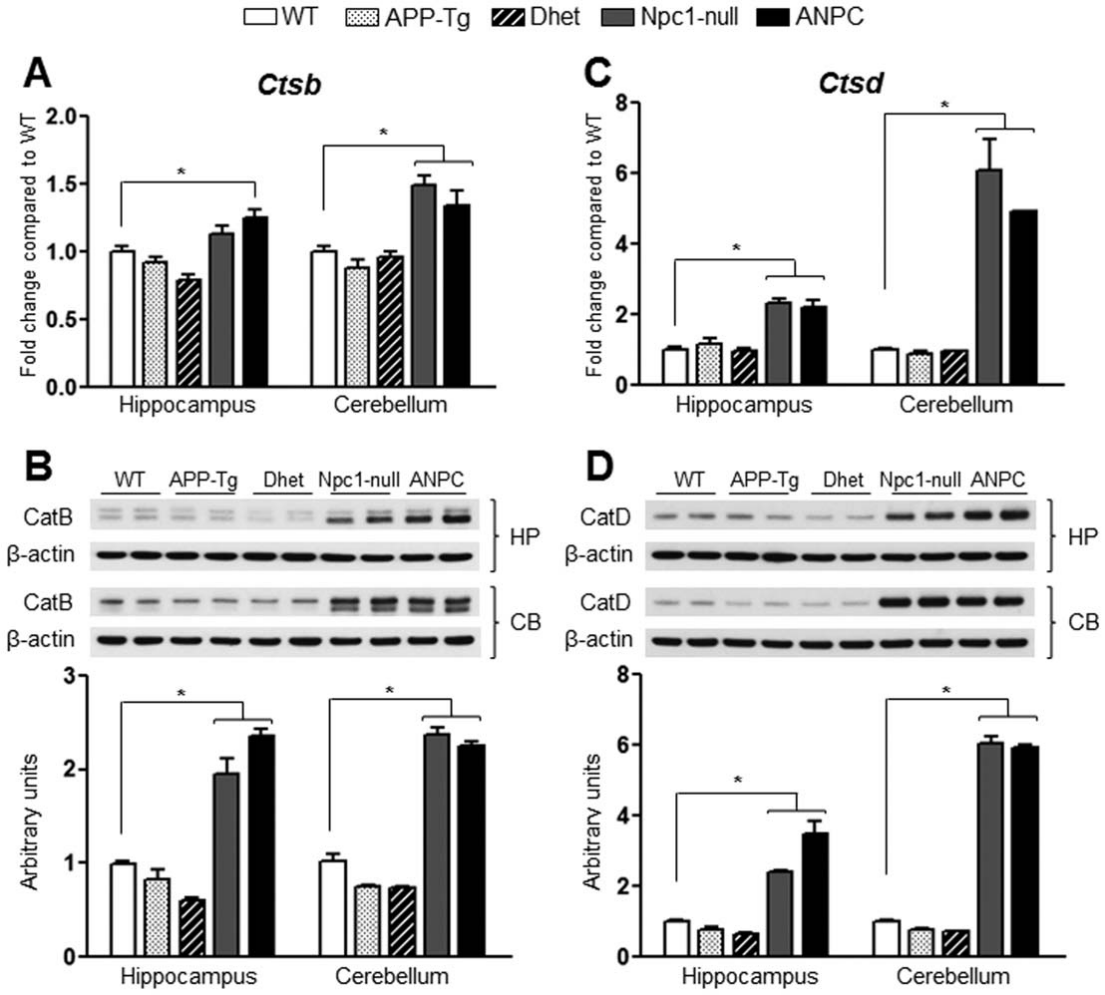
Transcript and protein expression levels of cathepsin B and cathepsin D in the hippocampus and cerebellum of five lines of mice. **A and C**, Histograms showing increased mRNA levels for *Ctsb* (encoding cathepsin B, A) and *Ctsd* (encoding cathepsin D, C) in the hippocampus and cerebellum of Npc1-null and ANPC mice compared with WT control mice as obtained using customized real-time RT-PCR array. **B and D,** Immunoblotting performed to validate data obtained by PCR arrays revealed increased levels of cathepsin B (B) and cathepsin D (D) in the hippocampus and cerebellum of Npc1-null and ANPC mice compared with the WT mice. APP-Tg and Dhet mice showed no alteration in transcript or protein expression levels of cathepsin B and cathepsin D compared with WT mice. The protein levels of cathepsin B and cathepsin D were normalized to the β-actin and the values (n = 4 animals/genotype) are expressed as means ± SEM. *, *p*<0.05. CatB, cathepsin B; CatD, cathepsin D.

**Figure 6:**
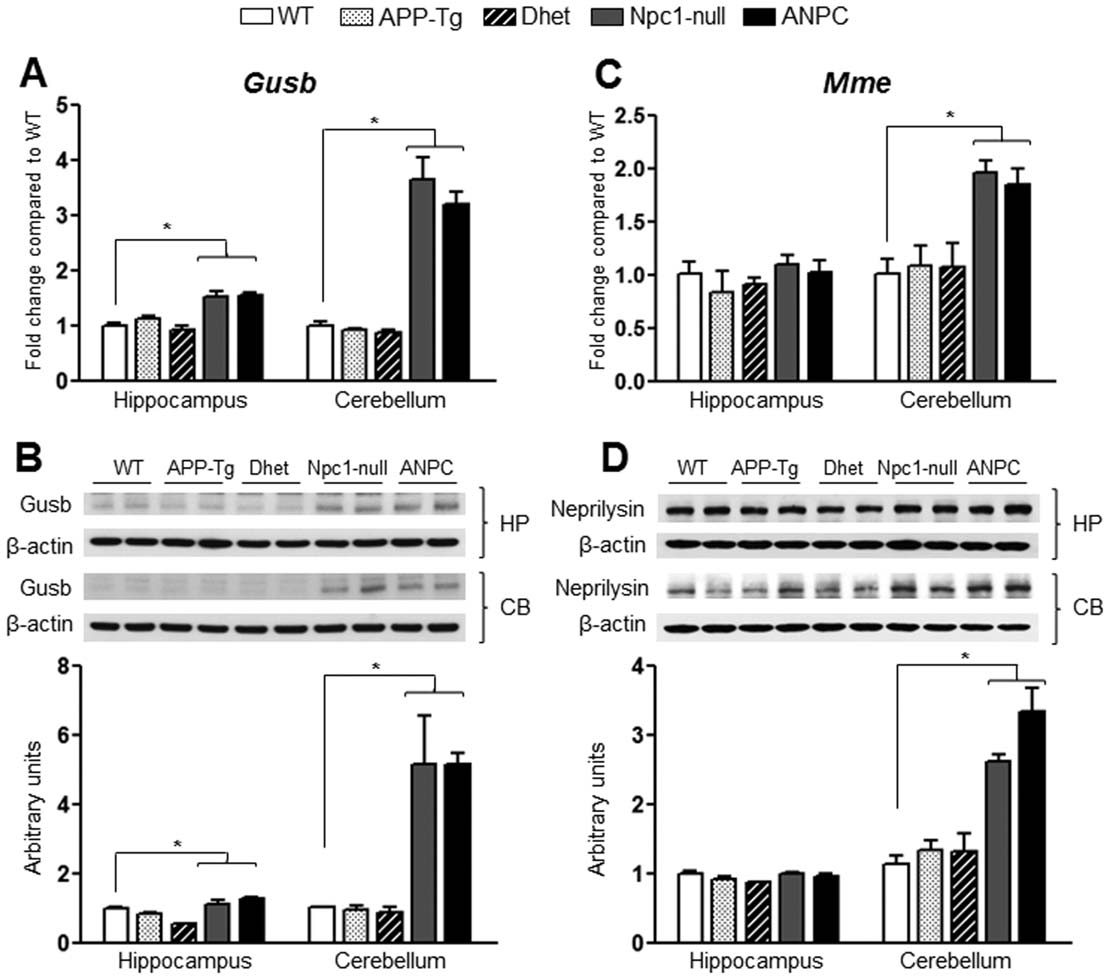
Transcript and protein expression levels of β-glucoronidase/Gusb and neprilysin/Mme in the hippocampus and cerebellum of five lines of mice. **A and C,** Histograms showing increased mRNA levels for *Gusb* (A) in both hippocampus and cerebellum and *Mme* (encoding neprilysin, C) in the cerebellum of Npc1-null and ANPC mice compared with WT control mice as obtained using customized real-time RT-PCR array. **B and D,** Immunoblotting performed to validate data obtained by PCR arrays revealed increased levels of Gusb (B) in both the hippocampus and cerebellum and neprilysin (D) in the cerebellum of Npc1-null and ANPC mice compared with WT mice. APP-Tg and Dhet mice showed no significant alteration in transcript or protein expression levels of Gusb (A, B) or neprilysin (C, D) compared with WT mice. The protein levels of Gusb and neprilysin were normalized to the β-actin and the values (n = 4 animals per genotype) are expressed as means ± SEM. *, *p*<0.05.

**Figure 7:**
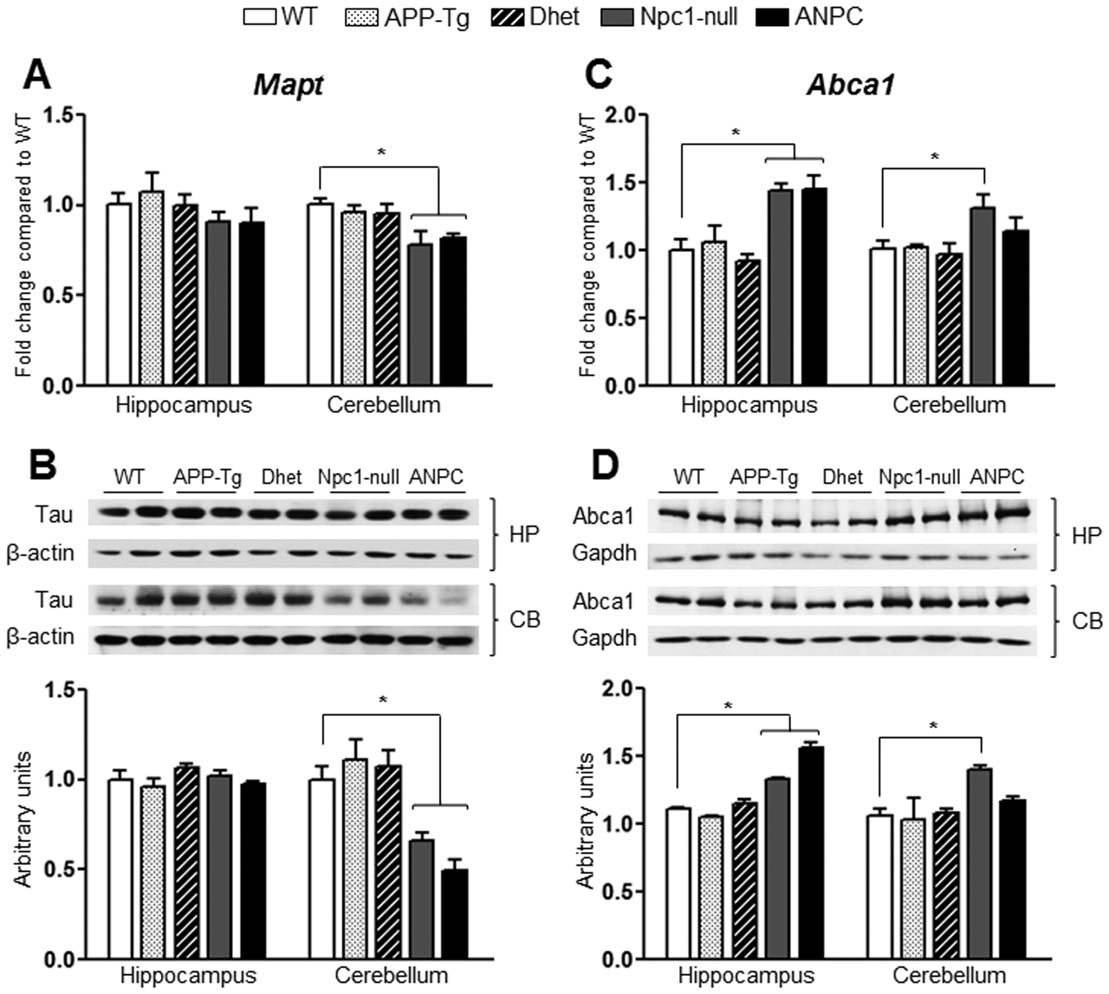
Transcript and protein expression levels of tau/Mapt and Abca1 in the hippocampus and cerebellum of five lines of mice. **A,** Histograms showing decreased *Mapt* mRNA (encoding tau) level in the cerebellum but not in hippocampus of Npc1-null and ANPC mice compared with WT mice as obtained using customized real-time RT-PCR array. **B,** Immunoblots and respective histograms validating the decreased levels of tau in the cerebellum of Npc1-null and ANPC mice compared with age-matched WT mice. C, Histograms showing increased *Abca1* mRNA level in the hippocampus of Npc1-null and ANPC mice and in the cerebellum of Npc1-null mice compared with WT as obtained using customized real-time RT-PCR array. D, Immunoblotting performed to validate data obtained by PCR arrays revealed significant up-regulations in the Abca1 protein level in Npc1-null and ANPC mice in the respective brain regions compared with WT mice. The protein levels of tau and Abca1 were normalized to those of β-actin and Gapdh respectively, and the values (n = 4 animals per genotype) are expressed as means ± SEM. *, *p*<0.05.

In order to gain molecular insights into the exacerbated neuropathological changes observed in ANPC mice, we analyzed the expression profiles of 86 selected genes involved in APP and Aβ metabolism, cholesterol homeostasis, intracellular vesicular trafficking and cell death mechanisms in the affected cerebellar and relatively spared hippocampal regions at 7-weeks of age (when distinct neuropathological and behavioral abnormalities are evident) from five different genotypes (i.e., WT, APP-Tg, Dhet, Npc1-null and ANPC mice) (Table 1). Our results of the real-time RT-PCR array analysis showed significant alterations in the relative expression of a wide-spectrum of transcripts in the brain regions of APP-Tg, Dhet, Npc1-null and ANPC mice compared to the WT controls (see Fig. 2). Complete list of differentially expressed genes with the respective fold-change in APP-Tg, Dhet, Npc1-null and ANPC mice compared to WT mice are provided in Tables S1–S8 in File S1. Intriguingly, ANPC and Npc1-null mice with the exception of some subtle differences, exhibited more or less similar changes, *albeit* the magnitude of alteration differs between the affected cerebellar *vs* relatively spared hippocampal regions (Fig. 2; Tables S1–S8 in File S1). The changes observed in APP-Tg and Dhet mice were limited to only a few transcripts mostly in the hippocampus (Fig. 3A, B), whereas Npc1-null and ANPC mice showed striking alterations in the expression profiles of a greater number of genes in both hippocampus and cerebellum compared to WT mice (Fig. 3C, D). Of the 86 genes evaluated, 14 transcripts (i.e., *Ctsb*, *Ctsd*, *Gusb*, *A2m*, *Npc2*, *Apoe*, *Mme*, *Clu*, *Plat*, *Plau*, *Cyp46a1*, *Srebf1*, *Pmaip1* and *Bid*) were significantly (*p*<0.05) up-regulated (Figs. 4,5,6) and 4 genes (i.e., *Anax6*, *Klc2*, *Aph1b* and *Mapt*) were significantly (*p*<0.05) down-regulated (Fig. 7) in the cerebellum of ANPC and Npc1-null mice compared to WT mice. In contrast to cerebellum, expression of only 6 genes (i.e, *Ctsd*, *Gusb*, *A2m*, *Npc2*, *Apoe* and *Abca1*) were significantly (*p*<0.05) up-regulated (Figs. 4,5,6,7) and one gene (i.e., *Tubb4*) is significantly (*p*<0.05) down-regulated in the hippocampus of ANPC and Npc1-null mice as compared with WT mice. Apart from the similarities, expression profiles of selected genes were differentially regulated in Npc1-null *vs* ANPC mice. For example, while the expression of *Abca1*, *Park2* and *Shisa5* genes were significantly (*p*<0.05) up-regulated in the cerebellum and that of *Dhcr24* down regulated in the hippocampus of Npc1-null mice as compared with WT mice, no such alteration was observed in ANPC mice (Fig. 7). With regards to ANPC mice, we observed down-regulation of *Cyp46a1*, *Rabggta* and *Kif1c* and up-regulation of *Ctsb* in the hippocampus compared to WT controls. However, expression of these genes was not significantly altered in Npc1-null mice (Fig. 5).

**Table 1:** List of selected genes for the customized real-time RT-PCR array.

NCBI Ref Seq^#^	Gene Symbol	Official Gene Name
***A. Selected genes related to APP/Aβ metabolism***		
NM_175628	*A2m*	Alpha-2-macroglobulin
NM_007399	*Adam10*	A disintegrin and metallopeptidase domain 10
NM_009615	*Adam17*	A disintegrin and metallopeptidase domain 17
NM_146104	*Aph1a*	Anterior pharynx defective 1a homolog (C. elegans)
NM_177583	*Aph1b*	Anterior pharynx defective 1b homolog (C. elegans)
NM_007467	*Aplp1*	Amyloid beta (A4) precursor-like protein 1
NM_011792	*Bace1*	Beta-site APP cleaving enzyme 1
NM_019517	*Bace2*	Beta-site APP-cleaving enzyme 2
NM_031156	*Ide*	Insulin degrading enzyme
NM_008604	*Mme*	Membrane metallo endopeptidase
NM_021607	*Ncstn*	Nicastrin
NM_008872	*Plat*	Plasminogen activator, tissue
NM_008873	*Plau*	Plasminogen activator, urokinase
NM_008877	*Plg*	Plasminogen
NM_008943	*Psen1*	Presenilin 1
NM_011183	*Psen2*	Presenilin 2
NM_025498	*Psenen*	Presenilin enhancer 2 homolog (C. elegans)
NM_013697	*Ttr*	Transthyretin
***B. Selected genes involved in cholesterol metabolism***		
NM_013454	*Abca1*	ATP-binding cassette, sub-family A (ABC1), member 1
NM_031884	*Abcg5*	ATP-binding cassette, sub-family G (WHITE), member 5
NM_009338	*Acat2*	Acetyl-Coenzyme A acetyltransferase 2
NM_009696	*Apoe*	Apolipoprotein E
NM_013492	*Clu*	Clusterin
NM_010010	*Cyp46a1*	Cytochrome P450, family 46, subfamily a, polypeptide 1
NM_053272	*Dhcr24*	24-dehydrocholesterol reductase
NM_010191	*Fdft1*	Farnesyl diphosphate farnesyl transferase 1
NM_134469	*Fdps*	Farnesyl diphosphate synthetase
NM_008255	*Hmgcr*	3-hydroxy-3-methylglutaryl-Coenzyme A reductase
NM_010700	*Ldlr*	Low density lipoprotein receptor
NM_008512	*Lrp1*	Low density lipoprotein receptor-related protein 1
NM_207242	*Npc1l1*	NPC1-like 1
NM_023409	*Npc2*	Niemann Pick type C2
NM_009473	*Nr1h2*	Nuclear receptor subfamily 1, group H, member 2
NM_001001144	*Scap*	SREBF chaperone
NM_009270	*Sqle*	Squalene epoxidase
NM_011480	*Srebf1*	Sterol regulatory element binding transcription factor 1
NM_033218	*Srebf2*	Sterol regulatory element binding factor 2
***C. Selected genes involved in intracellular trafficking***		
NM_013472	*Anxa6*	Annexin A6
NM_015740	*Bloc1s1*	Biogenesis of lysosome-related organelles complex-1, subunit 1
NM_021538	*Cope*	Coatomer protein complex, subunit epsilon
NM_007864	*Dlg4*	Discs, large homolog 4 (Drosophila)
NM_010064	*Dync1i2*	Dynein cytoplasmic 1 intermediate chain 2
NM_010515	*Igf2r*	Insulin-like growth factor 2 receptor
NM_153103	*Kif1c*	Kinesin family member 1C
NM_008451	*Klc2*	Kinesin light chain 2
NM_010684	*Lamp1*	Lysosomal-associated membrane protein 1
NM_010749	*M6pr*	Mannose-6-phosphate receptor, cation dependent
NM_010838	*Mapt*	Microtubule-associated protein tau
NM_025887	*Rab5a*	RAB5A, member RAS oncogene family
NM_009005	*Rab7*	RAB7, member RAS oncogene family
NM_019773	*Rab9*	RAB9, member RAS oncogene family
NM_145522	*Rabepk*	Rab9 effector protein with kelch motifs
NM_019519	*Rabggta*	Rab geranylgeranyl transferase, a subunit
NM_009294	*Stx4a*	Syntaxin 4A (placental)
NM_009305	*Syp*	Synaptophysin
NM_009451	*Tubb4*	Tubulin, beta 4
***D. Selected genes implicated in cell death/survival pathways***		
NM_009652	*Akt1*	Thymoma viral proto-oncogene 1
NM_026217	*Atg12*	Autophagy-related 12 (yeast)
NM_053069	*Atg5*	Autophagy-related 5 (yeast)
NM_028835	*Atg7*	Autophagy-related 7 (yeast)
NM_007527	*Bax*	Bcl2-associated X protein
NM_009741	*Bcl2*	B-cell leukemia/lymphoma 2
NM_019584	*Becn1*	Beclin 1, autophagy related
NM_007544	*Bid*	BH3 interacting domain death agonist
NM_133926	*Camk1*	Calcium/calmodulin-dependent protein kinase I
NM_007602	*Capn5*	Calpain 5
NM_009810	*Casp3*	Caspase 3
NM_009817	*Cast*	Calpastatin
NM_007668	*Cdk5*	Cyclin-dependent kinase 5
NM_007798	*Ctsb*	Cathepsin B
NM_009983	*Ctsd*	Cathepsin D
NM_010234	*Fos*	FBJ osteosarcoma oncogene
NM_019827	*Gsk3b*	Glycogen synthase kinase 3 beta
NM_010368	*Gusb*	Glucuronidase, beta
NM_010514	*Igf2*	Insulin-like growth factor 2
NM_026160	*Map1lc3b*	Microtubule-associated protein 1 light chain 3 beta
NM_011949	*Mapk1*	Mitogen-activated protein kinase 1
NM_011952	*Mapk3*	Mitogen-activated protein kinase 3
NM_016700	*Mapk8*	Mitogen-activated protein kinase 8
NM_016694	*Park2*	Parkinson disease (autosomal recessive, juvenile) 2, parkin
NM_181414	*Pik3c3*	Phosphoinositide-3-kinase, class 3
NM_008839	*Pik3ca*	Phosphatidylinositol 3-kinase, catalytic, alpha polypeptide
NM_023371	*Pin1*	Protein (peptidyl-prolyl cis/trans isomerase) NIMA-interacting 1
NM_021451	*Pmaip1*	Phorbol-12-myristate-13-acetate-induced protein 1
NM_011563	*Prdx2*	Peroxiredoxin 2
NM_025858	*Shisa5*	Shisa homolog 5 (*Xenopus laevis*)

### Validation of Altered Gene Expression Profiles by Western Blotting

**Figure 8:**
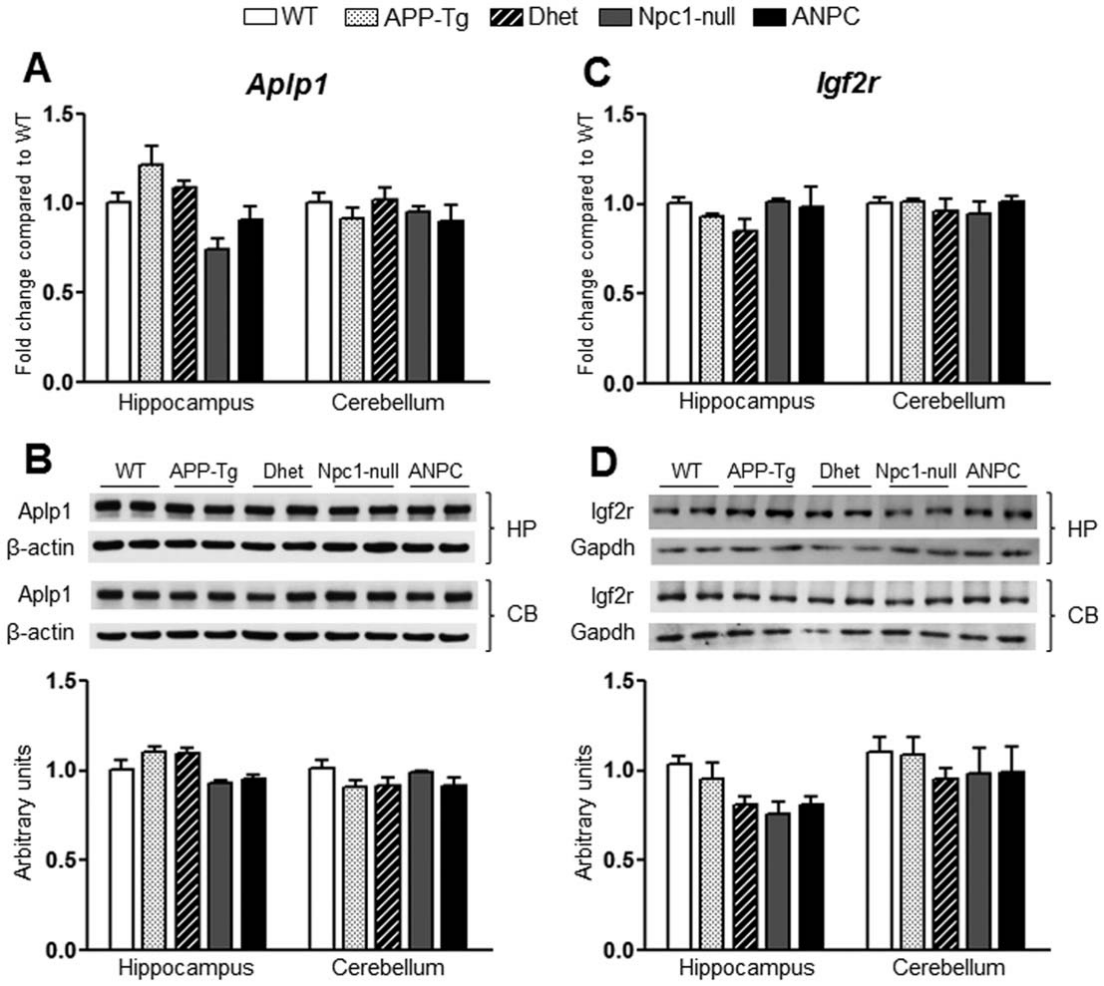
Transcript and protein expression levels of Aplp1 and Igf2r in the hippocampus and cerebellum of five lines of mice. **A and C**, Histograms showing no significant alteration in *Aplp1* (A) and *Igf2r* (C) mRNA levels in the hippocampus and cerebellum of APP-Tg, Dhet, Npc1-null and ANPC mice compared with WT mice as obtained using customized real-time RT-PCR array. **B and D,** Immunoblots and respective histograms showing no significant alteration in Aplp1 (B) and Igf2r (D) protein levels in the hippocampus or cerebellum of the different genotype combinations compared with WT mice consistent with the transcript levels. The protein levels of Aplp1 and Igf2r were normalized to the β-actin and GAPDH respectively, and the values (n = 4 animals per genotype) are expressed as means ± SEM. *, *p*<0.05.

**Figure 9:**
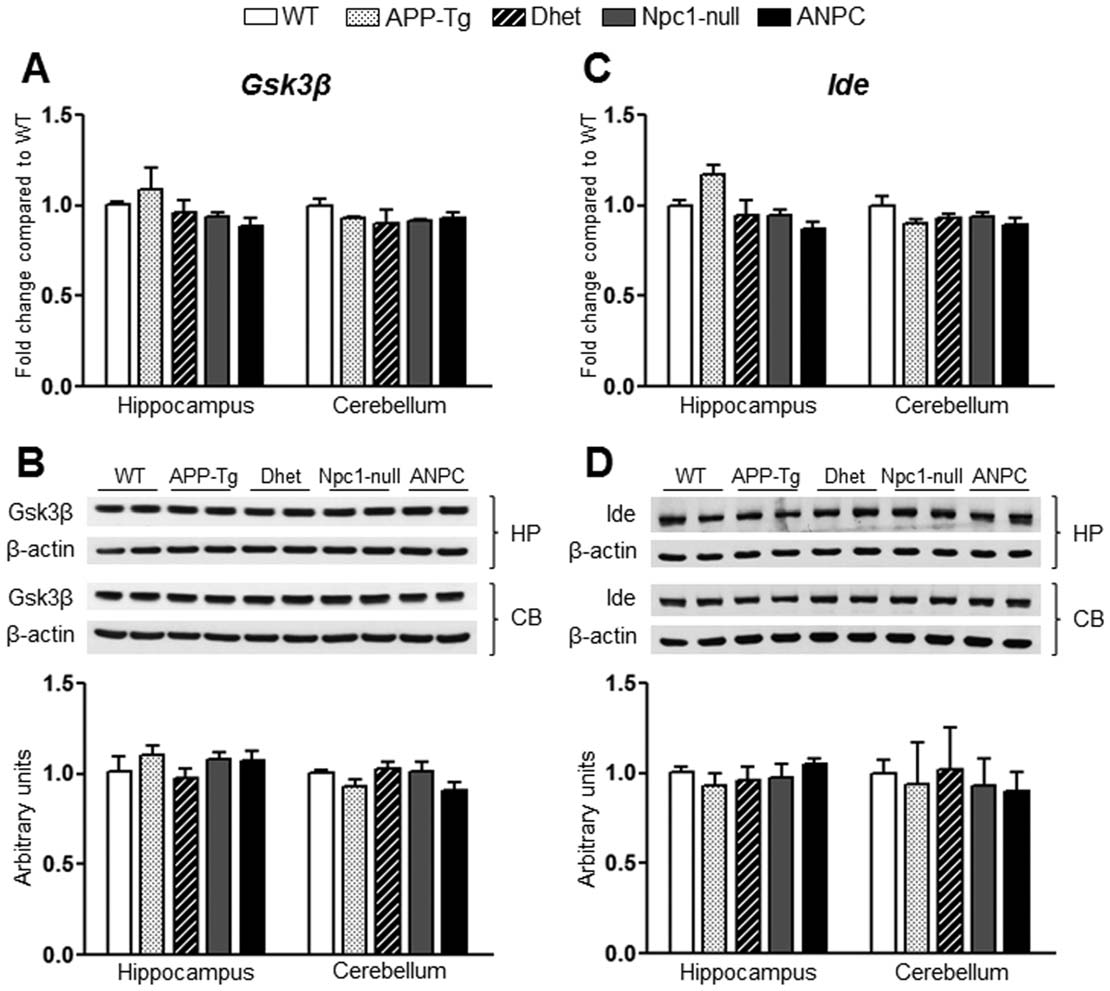
Transcript and protein expression levels of Gsk3β and Ide in the hippocampus and cerebellum of five lines of mice. **A and C**, Histograms showing no significant alteration in *Gsk3β* (A) and *Ide* (C) mRNA levels in the hippocampus and cerebellum of APP-Tg, Dhet, Npc1-null and ANPC mice compared with the WT mice as obtained using customized real-time RT-PCR array. **B and D,** Immunoblots and respective histograms showing no significant alteration in Gsk3β (B) and Ide (D) protein levels in the hippocampus or cerebellum of the different genotype combinations compared with the WT mice consistent with the transcript levels. The protein levels of Gsk3β and Ide were normalized to β-actin and the values (n = 4 animals per genotype) are expressed as means ± SEM. *, *p*<0.05.

To validate the observed changes in the gene expressions, we evaluated the steady-state protein levels of selected transcripts in both the hippocampus and cerebellum of all five lines of mice by immunoblot analysis. In keeping with up-regulated transcript levels, we observed significant (*p*<0.05) increase in the levels of Apoe, Npc2, cathepsin B, cathepsin D and β-glucoronidase (encoded by *Apoe*, *Npc2, Ctsb*, *Ctsd* and *Gusb*, respectively) in both the hippocampus and cerebellum of ANPC and Npc1-null mice compared with APP-Tg, Dhet and WT mice (Figs. 4,5,6). In addition, the levels of tau (encoded by *Mapt*) were significantly (*p*<0.05) decreased and the levels of neprilysin (encoded by *Mme*) were markedly increased in the cerebellum, but not in the hippocampus, of ANPC and Npc1-null mice compared to littermates of other genotypes (Figs. 6 and 7). Consistent with *Abca1* transcripts, the protein levels were significantly (*p*<0.05) enhanced in the hippocampus of Npc1-null and ANPC mice as well as in the cerebellum of Npc1-null mice compared to WT mice (Fig. 7). Interestingly, the levels of certain proteins such as Apoe and neprilysin, but not their transcripts, are significantly higher in ANPC than Npc1-null mice, suggesting potential translational control or post-translational modifications contributing to their steady-state protein levels (Figs. 4 and 6). As expected from the relative fold-change quantified from real-time RT-PCR array data, immunoblot analysis showed more prominent changes for proteins such as cathepsin D, neprilysin, tau and β-glucoronidase in the affected cerebellum than hippocampus in both ANPC and Npc1-null mice (Figs. 5,6,7). Consistent with unaltered transcript levels, no significant changes were evident in the levels of Bace1, Cdk-5 (encoded by *Bace1*, *Cdk-5*, respectively; data not shown), Aplp1, Gsk3β, Ide and Igf2r (encoded by *Aplp1*, *Gsk3β, Ide* and *Igf2r*, respectively; Figs. 8 and 9) either in the hippocampus or cerebellum of ANPC and Npc1-null mice compared with APP-Tg, Dhet and WT mice.

## Discussion

The present study demonstrates several genes as evident by real-time RT-PCR arrays are selectively altered in the brains of ANPC, Npc1-null, APP-Tg and Dhet mice compared to WT mouse brains. While the Npc1-null and ANPC mice showed marked alterations in the expression profiles of multiple genes, the changes in APP-Tg and Dhet mice were limited to only few genes in both the hippocampus and cerebellum compared to WT mice. Interestingly, ANPC and Npc1-null mice, with the exception of a few genes, exhibited more or less similar changes, although more genes were differentially expressed in the affected cerebellar region than the relatively spared hippocampal formation. The altered gene profiles were found to match with the corresponding alterations in the protein levels. Collectively, these results suggest that Npc1 deficiency can alter the expression of selected genes, which may be involved either directly or indirectly in NPC pathogenesis, whereas expression of mutant human APP in Npc1-null background appears to influence disease pathology by regulating the changes that are already altered by Npc1-deficiency rather instigating expression of additional genes.

Our analysis of gene profiles is validated by two lines of evidence. First, the genes that are known to be enhanced by Npc1-deficiency such as *Npc2*, *Abca1* and *Ctsb* were found to be up-regulated in our experiments [26]. Second, the altered genes profiles exhibited corresponding changes in the levels of proteins. Nevertheless, it is important to note that the absolute fold- changes in the level of a specific transcript need not be of high magnitude to have a significant consequence on cell physiology. Additionally, post-translational modification of proteins can have an important role in regulating the extent of neuropathology and the severity of the phenotype of a mouse line. Given the evidence from earlier reports [17], [27], it is likely that some of the changes observed in the present study, such as increased cathepsin B and cathespin D levels, may underlie the cause rather than consequence of disease pathology. However, it is of interest to note that the present study was carried out on 7-week-old mice which may restrict the effects of mutant APP in Npc1-null mice as increased levels and expression of Aβ-related pathology are not significantly apparent until 5–6-months of age in this line of APP-Tg mice [24].

### Cholesterol Metabolism

As a major component of the cell membrane, cholesterol plays a key role in fluidity and ion permeability which in turn regulates a multitude of vesicular trafficking steps and intracellular signaling events that are crucial for neuronal differentiation, growth and survival [28]. Cytoplasmic cholesterol, on the other hand, can serve as a precursor for steroid hormones, vitamin D and oxysterols [29]. The special need of cholesterol for normal neuronal function is apparent from two distinct lines of evidence i) brain contains the highest amount of total cholesterol/gm tissue in the body [30] and ii) the cholesterol content of the brain is derived primarily from *de novo* synthesis [30], [31]. Under normal conditions, brain cholesterol level is tightly regulated by a number of mechanisms including synthesis, transport, uptake, storage and efflux of cholesterol. At the transcriptional level, sterol regulatory element-binding proteins regulate the expression profiles of multiple genes that are involved in monitoring the synthesis and uptake of cholesterol, fatty acids and phoshpholipids [32]. With adequate cholesterol in the endoplasmic reticulum, genes involved in cholesterol synthesis and uptake are not activated by sterol regulatory element-binding proteins. Npc1-deficient cells do not respond to the accumulation of unesterified cholesterol in the endosomal-lysosomal system, thus LDL receptor expression is not down-regulated and uptake of LDL-mediated cholesterol continues [33]. Our real-time RT-PCR array data reflect the dysregulation of cholesterol metabolism as we observed an up-regulation of *Apoe*, *Clu*, *Cyp46a1*, *Srebf1* and *Npc2* in the cerebellum and *Apoe*, *Abca1* and *Npc2* in the hippocampus of both Npc1-null and ANPC mice. There is evidence of selective up-regulation of *Abca1* in the cerebellum and down-regulation of *Dhcr24* in the hippocampus of Npc1-null mice. Consistent with the transcripts levels, we observed up-regulation of Abca1, Npc2 and Apoe in the cerebellum as well as hippocampus of ANPC and Npc1-null mice compared to WT mice by immunoblot analysis. The hippocampus of APP-Tg mice also showed increased expression of *Acat2*, *Fdps*, *Sqle* and *Dhcr24*, which may relate to alterations in the intracellular cholesterol homeostasis as a consequence of APP overexpression.

### APP and Aβ Metabolisms

A number of recent studies have shown that NPC disease exhibits some striking parallels with AD pathology including i) the presence of tau-positive neurofibrillary tangles [4], [5], ii) the influence of ε4 isoform APOE in promoting disease pathology [6], [34], and iii) endosomal abnormalities associated with the accumulation of cleaved APP derivatives and/or Aβ peptides in vulnerable neurons [11], [35]. However, consistent with previous analysis of NPC1-deficient human fibroblasts [26] and Npc1-deficient mouse cells [36], we did not observe any difference in the mRNA profiles of α-secretase (i.e., *Adam 10* and *Adam 17*), β-secretase (*Bace1* and *Bace2*) or most components of the γ-secretase complex (i.e., *Psen1*, *Psen2*, *Nicastrin*, *Pen2* and *Aph1a*) either in Npc1-null or ANPC mouse brains compared to WT mice. Only *Aph1b* showed down-regulation in the cerebellum of Npc1-null and ANPC mice, the significance of which remains to be established. At the protein level, no alteration was evident in the components of APP processing pathways, except increased levels of nicastrin, which may relate to activity of the γ-secretase complex or other functions of this protein in the cells [37], [38]. Interestingly, the levels of transcripts (i.e, *A2m*, *Plat*, *Plau* and *Mme*) and some of the corresponding proteins (i.e., neprilysin), which are known to be involved in the clearance of Aβ peptides [39], [40], were significantly up-regulated in the cerebellum of Npc1-null and ANPC mice. These results suggest that Npc1 deficiency may influence the clearance of Aβ peptides. However, we did not observe an alteration in the expression of *Ide*, which codes for one of the major enzymes involved in degradation of extracellular Aβ, either in ANPC or Npc1-null mice. Thus, it remains to be determined whether increased expression of *A2m*, *Plat*, *Plau* and *Mme* is directly associated with degradation of Aβ or other proteins in Npc1-null and ANPC mice.

The formation of neurofibrillary tangles resulting from phosphorylation of tau protein, encoded by the *Mapt* gene, has been implicated in the loss of neurons in many taupathies including NPC and AD [41]. Our results showed down-regulation of *Mapt* transcript in the cerebellum but not in the hippocampus of ANPC and Npc1-null mice as compared with WT mice. This corresponds rather well with the steady-state levels of tau protein observed by Western blot analysis. Since partial or complete loss of tau expression can reduce the lifespan and exacerbate pathology [42] and inhibition of tau phosphorylation can attenuate the phenotype [43] in Npc1-null mice, it is likely that alterations in total tau levels in the cerebellum of ANPC and Npc1-null mice may be involved in the loss of neurons and the development of pathology associated with these mice.

### Altered Trafficking

Cholesterol accumulation in NPC1-deficient cells has been shown to interfere with the transport of proteins between various cellular compartments. Consequently, proteins involved in membrane trafficking including those regulating biogenesis/function of lysosomes are up-regulated to compensate for the defects [44], [45]. In keeping with these data, we observed a significant increase in the expression of genes encoding lysosomal enzymes such as *Ctsb*, *Ctsd* and *Gusb*, both at transcript and protein levels, in the Npc1-null and ANPC mouse brains. This is consistent with earlier studies, which reported elevated levels of cathepsin B and cathepsin D in NPC1-deficient cells as well as Npc1-null and ANPC mouse brains [17], [23], [26], [46], [47]. These enzymes not only mediate the clearance of proteins but also regulate neuronal viability following their release into the cytosol. However, unlike the previous studies [26] we did not observe alterations in the levels of mRNA encoding *Igf2r*, which is involved in the trafficking of the lysosomal enzymes, either in Npc1-null or ANPC mouse brains. Additionally, we did not detect alterations in the expression of transcripts encoding Rab GTPase such as *Rab5*, *Rab7* and *Rab9* that are known to act as general regulators of membrane trafficking in the endosomal pathway [48], [49]. Other components involved in the vesicular movement such as *Klc2*, *Kif1c* and *Anx6* were differentially down-regulated in the Npc1-null and ANPC mice, but not in APP-Tg or Dhet mouse brains, possibly as a consequence of the defects triggered by Npc1 deficiency.

### Cell Death/survival Pathways

Although APP-Tg mouse model used in our study does not exhibit any overt loss of neurons [24], [25], there is evidence of neuronal loss in the cerebellum of Npc1-null mice [18], [20]. Additionally, we showed that ANPC mice exhibited more severe loss of neurons than Npc1-null mice thus suggesting that overexpression of APP may exacerbate neurodegeneration [23]. At present, the mechanisms underlying selective loss of neurons remain unclear as events related to both apoptosis and autophagy have been observed in Npc1-null mouse brains [2], [47], [50], [51]. The results of our study did not reveal any alterations in the expression of genes such as *Atg5*, *Atg7*, *Atg12*, *Becn1*, *Bcl2*, *Casp3* or *Bax*, which are known to regulate autophagy or apoptosis pathways, either in Npc1-null or ANPC mouse brains. The affected cerebellar region, however, showed a marked up-regulation of *Bid* and *Pmaip1* in both Npc1-null and ANPC mice, whereas expression of *Shisa5* was up-regulated only in Npc1-null mice. Although significance of the differential expression of these genes remains unclear, we have recently reported that increased cytosolic levels of lysosomal enzymes resulting from lysosomal destabilization, such as cathepsin B and cathepsin D, as well as altered levels, phosphorylation and cleavage of tau protein, may be involved in triggering cell death *via* a caspase-dependent pathway in Npc1-null and ANPC mouse brains [17], [23]. This is consistent with the up-regulation of *Ctsb* and *Ctsd*, and down-regulation of *Mapt* transcripts and their corresponding protein levels observed in the cerebellum of Npc1-null and ANPC mouse brains compared with WT mice.

### Conclusions

The present study reveals that gene expression profile is differentially altered in APP-Tg, Dhet, Npc1-null and ANPC mouse brains when compared to WT mice. These changes are found to be more striking in Npc1-null and ANPC mice, which exhibit more severe pathology than APP-Tg or Dhet mice. Additionally, the changes in Npc1-null and ANPC mice were more pronounced in the affected cerebellar region than the relatively spared hippocampus. Further investigations on the profiles of altered genes and the functional characterization of corresponding proteins will reveal important clues about their potential role in the pathogenesis of NPC and AD.

## Supporting Information

File S1 Gene expression profiles in the hippocampus and cerebellum of APP-Tg, Dhet, Npc1-null and ANPC mice compared to the WT mice as studied using real-time RT-PCR arrays.
(DOC)
