# Supplementary material for: Alterations in Gene Expression in Mutant Amyloid Precursor Protein Transgenic Mice Lacking Niemann-Pick Type C1 Protein
Source: PLoS One. 2013 Jan 29;8(1):e54605. doi: 10.1371/journal.pone.0054605 (PMC3558508; doi:10.1371/journal.pone.0054605)
Supplement: File S1 — Gene expression profiles in the hippocampus and cerebellum of APP-Tg, Dhet, Npc1-null and ANPC mice compared to the WT mice as studied using real-time RT-PCR arrays. (DOC) [file pone.0054605.s001.doc]

**Table S1.** Expression profiles of genes related to APP/Aβ metabolism in the hippocampus of APP-Tg, Dhet, Npc1-null and ANPC compared to the wild-type mice

| **Gene Symbol** | **APP-Tg** | | **Dhet** | | **Npc1-null** | | **ANPC** | |
| --- | --- | --- | --- | --- | --- | --- | --- | --- |
|  | **Fold Regulation** | **p-value** | **Fold Regulation** | **p-value** | **Fold Regulation** | **p-value** | **Fold Regulation** | **p-value** |
| ***A2m*** | 1.45 | 0.073 | -1.07 | 0.640 | 1.84 | 0.001 | 2.08 | 0.010 |
| ***Adam10*** | -1.03 | 0.789 | -1.05 | 0.470 | -1.14 | 0.044 | -1.15 | 0.103 |
| ***Adam17*** | -1.06 | 0.693 | -1.35 | 0.124 | -1.25 | 0.218 | -1.39 | 0.118 |
| ***Aph1a*** | 1.01 | 0.894 | -1.05 | 0.660 | 1.03 | 0.881 | 1.05 | 0.766 |
| ***Aph1b*** | 1.12 | 0.417 | 1.10 | 0.379 | -1.20 | 0.204 | 1.02 | 0.898 |
| ***Aplp1*** | 1.20 | 0.129 | 1.09 | 0.248 | -1.32 | 0.108 | -1.11 | 0.359 |
| ***Bace1*** | -1.11 | 0.318 | -1.09 | 0.226 | -1.12 | 0.153 | -1.23 | 0.064 |
| ***Bace2*** | 1.12 | 0.357 | -1.31 | 0.189 | 1.26 | 0.053 | -1.13 | 0.243 |
| ***Ide*** | 1.17 | 0.024 | -1.06 | 0.570 | -1.06 | 0.203 | -1.15 | 0.038 |
| ***Mme*** | -1.39 | 0.470 | -1.10 | 0.451 | 1.08 | 0.600 | 1.01 | 0.948 |
| ***Ncstn*** | -1.07 | 0.561 | -1.17 | 0.090 | 1.04 | 0.448 | 1.01 | 0.834 |
| ***Plat*** | 1.21 | 0.064 | 1.04 | 0.756 | 1.03 | 0.833 | 1.10 | 0.306 |
| ***Plau*** | 1.03 | 0.788 | 1.04 | 0.755 | 1.37 | 0.163 | 1.54 | 0.061 |
| ***Plg*** | 1.12 | 0.429 | -1.15 | 0.397 | -1.15 | 0.678 | -1.19 | 0.360 |
| ***Psen1*** | -1.05 | 0.618 | -1.14 | 0.302 | -1.11 | 0.247 | -1.18 | 0.258 |
| ***Psen2*** | 1.14 | 0.361 | 1.06 | 0.642 | 1.07 | 0.519 | 1.10 | 0.421 |
| ***Psenen*** | 1.06 | 0.532 | 1.03 | 0.518 | -1.11 | 0.068 | -1.04 | 0.452 |
| ***Ttr*** | -2.47 | 0.306 | -2.50 | 0.342 | 3.99 | 0.975 | 1.34 | 0.416 |

**Table S2.** Expression profiles of genes related to APP/Aβ metabolism in the cerebellum of APP-Tg, Dhet, Npc1-null and ANPC compared to the wild-type mice

| **Gene Symbol** | **APP-Tg** | | **Dhet** | | **Npc1-null** | | **ANPC** | |
| --- | --- | --- | --- | --- | --- | --- | --- | --- |
|  | **Fold Regulation** | **p-value** | **Fold Regulation** | **p-value** | **Fold Regulation** | **p-value** | **Fold Regulation** | **p-value** |
| ***A2m*** | 1.02 | 0.787 | 1.03 | 0.680 | 1.43 | 0.009 | 1.64 | 0.002 |
| ***Adam10*** | 1.03 | 0.915 | -1.03 | 0.842 | -1.29 | 0.110 | -1.26 | 0.177 |
| ***Adam17*** | -1.15 | 0.266 | -1.19 | 0.234 | -1.16 | 0.287 | -1.33 | 0.236 |
| ***Aph1a*** | 1.00 | 0.965 | -1.02 | 0.832 | 1.04 | 0.713 | -1.02 | 0.753 |
| ***Aph1b*** | -1.00 | 0.981 | 1.00 | 0.945 | -1.24 | 0.019 | -1.27 | 0.016 |
| ***Aplp1*** | -1.11 | 0.297 | 1.01 | 0.886 | -1.05 | 0.436 | -1.07 | 0.650 |
| ***Bace1*** | 1.01 | 0.915 | 1.00 | 0.987 | -1.22 | 0.062 | -1.17 | 0.072 |
| ***Bace2*** | -1.23 | 0.104 | -1.15 | 0.311 | 1.06 | 0.598 | 1.01 | 0.910 |
| ***Ide*** | -1.11 | 0.113 | -1.08 | 0.197 | -1.07 | 0.274 | -1.10 | 0.241 |
| ***Mme*** | -1.17 | 0.572 | 1.05 | 0.822 | 1.96 | 0.006 | 1.99 | 0.003 |
| ***Ncstn*** | -1.02 | 0.933 | 1.00 | 0.999 | -1.00 | 0.985 | -1.11 | 0.180 |
| ***Plat*** | 1.11 | 0.399 | 1.12 | 0.423 | 1.51 | 0.008 | 1.69 | 0.001 |
| ***Plau*** | 1.19 | 0.445 | 1.15 | 0.529 | 13.82 | 0.000 | 10.50 | 0.000 |
| ***Plg*** | -1.19 | 0.703 | -1.04 | 0.786 | -1.05 | 0.731 | 1.20 | 0.326 |
| ***Psen1*** | 1.02 | 0.800 | -1.07 | 0.378 | -1.11 | 0.165 | -1.18 | 0.031 |
| ***Psen2*** | 1.05 | 0.659 | 1.01 | 0.996 | 1.04 | 0.702 | -1.08 | 0.650 |
| ***Psenen*** | -1.00 | 0.949 | -1.04 | 0.610 | -1.00 | 0.987 | -1.15 | 0.186 |
| ***Ttr*** | -6.75 | 0.961 | -1.48 | 0.928 | -1.90 | 0.323 | -37.36 | 0.039 |

**Table S3.** Expression profiles of genes involved in cholesterol metabolism in the hippocampus of APP-Tg, Dhet, Npc1-null and ANPC compared to the wild-type mice

| **Gene Symbol** | **APP-Tg** | | **Dhet** | | **Npc1-null** | | **ANPC** | |
| --- | --- | --- | --- | --- | --- | --- | --- | --- |
|  | **Fold Regulation** | **p-value** | **Fold Regulation** | **p-value** | **Fold Regulation** | **p-value** | **Fold Regulation** | **p-value** |
| ***Abca1*** | 1.04 | 0.724 | -1.09 | 0.348 | 1.43 | 0.003 | 1.44 | 0.012 |
| ***Abcg5*** | 1.26 | 0.489 | -1.45 | 0.137 | 1.35 | 0.403 | -1.23 | 0.342 |
| ***Acat2*** | 1.50 | 0.021 | 1.23 | 0.235 | 1.10 | 0.407 | 1.28 | 0.063 |
| ***Apoe*** | 1.12 | 0.600 | 1.21 | 0.331 | 1.60 | 0.048 | 1.76 | 0.048 |
| ***Clu*** | 1.10 | 0.466 | -1.04 | 0.699 | 1.12 | 0.197 | 1.15 | 0.155 |
| ***Cyp46a1*** | -1.42 | 0.006 | -1.24 | 0.010 | -1.15 | 0.135 | -1.27 | 0.039 |
| ***Dhcr24*** | 1.34 | 0.011 | 1.31 | 0.02 | -1.23 | 0.013 | -1.03 | 0.819 |
| ***Fdft1*** | 1.26 | 0.02 | 1.39 | 0.019 | 1.01 | 0.885 | 1.33 | 0.096 |
| ***Fdps*** | 1.51 | 0.047 | 1.31 | 0.179 | -1.06 | 0.607 | 1.11 | 0.376 |
| ***Hmgcr*** | 1.42 | 0.071 | 1.12 | 0.314 | -1.15 | 0.450 | 1.01 | 0.836 |
| ***Ldlr*** | 1.57 | 0.081 | 1.41 | 0.079 | -1.00 | 0.925 | -1.00 | 0.819 |
| ***Lrp1*** | 1.11 | 0.319 | 1.04 | 0.716 | 1.23 | 0.169 | 1.20 | 0.176 |
| ***Npc1l1*** | -1.66 | 0.079 | -1.46 | 0.173 | -1.30 | 0.199 | 1.06 | 0.720 |
| ***Npc2*** | 1.00 | 0.905 | -1.04 | 0.477 | 1.42 | 0.001 | 1.50 | 0.002 |
| ***Nr1h2*** | 1.21 | 0.051 | -1.10 | 0.349 | -1.14 | 0.250 | -1.01 | 0.994 |
| ***Scap*** | 1.08 | 0.600 | -1.10 | 0.413 | -1.01 | 0.863 | 1.01 | 0.944 |
| ***Sqle*** | 1.53 | 0.037 | 1.34 | 0.035 | 1.05 | 0.709 | 1.12 | 0.396 |
| ***Srebf1*** | -1.15 | 0.115 | -1.32 | 0.006 | 1.01 | 0.909 | -1.06 | 0.471 |
| ***Srebf2*** | 1.17 | 0.180 | 1.18 | 0.099 | -1.01 | 0.952 | -1.04 | 0.842 |

**Table S4.** Expression profiles of genes involved in cholesterol metabolism in the cerebellum of APP-Tg, Dhet, Npc1-null and ANPC compared to the wild-type mice

| **Gene Symbol** | **APP-Tg** | | **Dhet** | | **Npc1-null** | | **ANPC** | |
| --- | --- | --- | --- | --- | --- | --- | --- | --- |
|  | **Fold Regulation** | **p-value** | **Fold Regulation** | **p-value** | **Fold Regulation** | **p-value** | **Fold Regulation** | **p-value** |
| ***Abca1*** | 1.02 | 0.781 | -1.04 | 0.738 | 1.34 | 0.043 | 1.18 | 0.402 |
| ***Abcg5*** | -1.74 | 0.144 | -1.15 | 0.826 | -1.11 | 0.693 | -1.38 | 0.355 |
| ***Acat2*** | -1.09 | 0.484 | 1.01 | 0.922 | 1.21 | 0.253 | 1.19 | 0.406 |
| ***Apoe*** | -1.20 | 0.425 | 1.09 | 0.770 | 2.43 | 0.012 | 2.10 | 0.013 |
| ***Clu*** | -1.04 | 0.763 | -1.02 | 0.799 | 1.47 | 0.012 | 1.43 | 0.037 |
| ***Cyp46a1*** | 1.04 | 0.608 | 1.18 | 0.094 | 1.23 | 0.040 | 1.38 | 0.032 |
| ***Dhcr24*** | -1.04 | 0.513 | 1.04 | 0.612 | 1.16 | 0.141 | 1.20 | 0.069 |
| ***Fdft1*** | -1.08 | 0.103 | 1.02 | 0.667 | 1.10 | 0.275 | 1.20 | 0.074 |
| ***Fdps*** | -1.08 | 0.499 | -1.02 | 0.802 | 1.11 | 0.379 | 1.11 | 0.466 |
| ***Hmgcr*** | -1.17 | 0.049 | -1.12 | 0.060 | -1.07 | 0.455 | 1.03 | 0.648 |
| ***Ldlr*** | -1.19 | 0.059 | -1.19 | 0.112 | -1.10 | 0.241 | -1.00 | 0.838 |
| ***Lrp1*** | 1.11 | 0.459 | 1.06 | 0.706 | 1.12 | 0.391 | 1.17 | 0.286 |
| ***Npc1l1*** | -1.37 | 0.228 | -1.21 | 0.499 | 1.05 | 0.788 | 1.37 | 0.377 |
| ***Npc2*** | -1.06 | 0.647 | -1.06 | 0.671 | 2.03 | 0.000 | 1.65 | 0.039 |
| ***Nr1h2*** | -1.02 | 0.883 | 1.02 | 0.865 | -1.13 | 0.238 | -1.10 | 0.490 |
| ***Scap*** | -1.01 | 0.786 | -1.06 | 0.345 | -1.06 | 0.377 | -1.06 | 0.595 |
| ***Sqle*** | -1.05 | 0.425 | 1.00 | 0.906 | 1.02 | 0.746 | 1.19 | 0.018 |
| ***Srebf1*** | -1.10 | 0.334 | -1.06 | 0.514 | 1.26 | 0.009 | 1.30 | 0.022 |
| ***Srebf2*** | -1.00 | 0.961 | -1.02 | 0.823 | -1.05 | 0.604 | -1.03 | 0.755 |

**Table S5.** Expression profiles of genes involved in intracellular trafficking in the hippocampus of APP-Tg, Dhet, Npc1-null and ANPC compared to the wild-type mice

| **Gene Symbol** | **APP-Tg** | | **Dhet** | | **Npc1-null** | | **ANPC** | |
| --- | --- | --- | --- | --- | --- | --- | --- | --- |
|  | **Fold Regulation** | **p-value** | **Fold Regulation** | **p-value** | **Fold Regulation** | **p-value** | **Fold Regulation** | **p-value** |
| ***Anxa6*** | -1.00 | 0.956 | 1.01 | 0.902 | -1.18 | 0.049 | -1.17 | 0.017 |
| ***Bloc1s1*** | 1.21 | 0.083 | 1.01 | 0.856 | 1.02 | 0.688 | -1.01 | 0.934 |
| ***Cope*** | 1.20 | 0.053 | -1.02 | 0.901 | -1.00 | 0.995 | 1.02 | 0.818 |
| ***Dlg4*** | -1.11 | 0.561 | 1.07 | 0.600 | -1.05 | 0.745 | -1.16 | 0.263 |
| ***Dync1i2*** | 1.05 | 0.350 | -1.11 | 0.271 | -1.17 | 0.064 | -1.21 | 0.078 |
| ***Igf2r*** | -1.07 | 0.145 | -1.19 | 0.082 | 1.01 | 0.807 | -1.03 | 0.884 |
| ***Kif1c*** | -1.30 | 0.070 | -1.21 | 0.126 | -1.25 | 0.19 | -1.42 | 0.038 |
| ***Klc2*** | -1.12 | 0.316 | -1.06 | 0.465 | -1.16 | 0.051 | -1.23 | 0.096 |
| ***Lamp1*** | 1.16 | 0.164 | -1.02 | 0.785 | 1.07 | 0.362 | 1.04 | 0.506 |
| ***M6pr*** | 1.09 | 0.199 | -1.01 | 0.971 | -1.02 | 0.889 | -1.01 | 1.000 |
| ***Mapt*** | 1.00 | 0.926 | -1.07 | 0.511 | -1.16 | 0.139 | -1.23 | 0.092 |
| ***Rab5a*** | 1.06 | 0.597 | -1.23 | 0.243 | -1.11 | 0.233 | -1.06 | 0.523 |
| ***Rab7*** | 1.07 | 0.527 | 1.05 | 0.609 | 1.04 | 0.574 | 1.08 | 0.433 |
| ***Rab9*** | 1.19 | 0.067 | 1.03 | 0.661 | -1.04 | 0.617 | -1.02 | 0.861 |
| ***Rabepk*** | 1.21 | 0.170 | 1.20 | 0.054 | -1.03 | 0.875 | -1.09 | 0.563 |
| ***Rabggta*** | -1.06 | 0.295 | -1.11 | 0.093 | -1.12 | 0.028 | -1.23 | 0.001 |
| ***Stx4a*** | 1.09 | 0.412 | 1.00 | 0.985 | -1.06 | 0.552 | -1.11 | 0.407 |
| ***Syp*** | -1.11 | 0.361 | -1.02 | 0.894 | -1.09 | 0.426 | -1.13 | 0.226 |
| ***Tubb4*** | -1.00 | 0.969 | -1.05 | 0.488 | -1.45 | 0.001 | -1.60 | 0.000 |

**Table S6.** Expression profiles of genes involved in intracellular trafficking in the cerebellum of APP-Tg, Dhet, Npc1-null and ANPC compared to the wild-type mice

| **Gene Symbol** | **APP-Tg** | | **Dhet** | | **Npc1-null** | | **ANPC** | |
| --- | --- | --- | --- | --- | --- | --- | --- | --- |
|  | **Fold Regulation** | **p-value** | **Fold Regulation** | **p-value** | **Fold Regulation** | **p-value** | **Fold Regulation** | **p-value** |
| ***Anxa6*** | 1.00 | 0.995 | -1.04 | 0.634 | -1.25 | 0.028 | -1.37 | 0.022 |
| ***Bloc1s1*** | 1.11 | 0.499 | 1.08 | 0.643 | 1.03 | 0.913 | -1.03 | 0.805 |
| ***Cope*** | 1.00 | 0.993 | -1.05 | 0.222 | -1.00 | 0.988 | -1.07 | 0.353 |
| ***Dlg4*** | 1.01 | 0.901 | 1.02 | 0.782 | -1.11 | 0.349 | -1.01 | 0.957 |
| ***Dync1i2*** | -1.11 | 0.003 | -1.08 | 0.243 | -1.10 | 0.010 | -1.07 | 0.550 |
| ***Igf2r*** | 1.02 | 0.734 | -1.04 | 0.633 | -1.07 | 0.465 | 1.01 | 0.803 |
| ***Kif1c*** | -1.10 | 0.460 | -1.14 | 0.458 | 1.15 | 0.304 | -1.19 | 0.236 |
| ***Klc2*** | 1.06 | 0.463 | 1.02 | 0.783 | -1.28 | 0.002 | -1.27 | 0.015 |
| ***Lamp1*** | -1.12 | 0.147 | -1.09 | 0.174 | 1.18 | 0.091 | 1.12 | 0.379 |
| ***M6pr*** | 1.00 | 0.998 | 1.05 | 0.735 | 1.06 | 0.670 | 1.21 | 0.194 |
| ***Mapt*** | -1.04 | 0.476 | -1.06 | 0.494 | -1.29 | 0.03 | -1.24 | 0.013 |
| ***Rab5a*** | -1.04 | 0.463 | -1.11 | 0.192 | -1.08 | 0.310 | 1.03 | 0.669 |
| ***Rab7*** | 1.05 | 0.547 | 1.00 | 0.982 | -1.60 | 0.180 | 1.08 | 0.292 |
| ***Rab9*** | -1.01 | 0.743 | -1.07 | 0.083 | -1.13 | 0.117 | -1.08 | 0.255 |
| ***Rabepk*** | -1.06 | 0.634 | -1.03 | 0.760 | -1.06 | 0.536 | -1.12 | 0.432 |
| ***Rabggta*** | -1.01 | 0.729 | -1.01 | 0.813 | -1.02 | 0.547 | 1.02 | 0.682 |
| ***Stx4a*** | -1.08 | 0.403 | -1.07 | 0.356 | -1.04 | 0.628 | -1.12 | 0.243 |
| ***Syp*** | -1.03 | 0.798 | -1.01 | 0.909 | -1.12 | 0.171 | -1.05 | 0.635 |
| ***Tubb4*** | 1.03 | 0.761 | -1.07 | 0.435 | -1.14 | 0.196 | -1.20 | 0.133 |

**Table S7.** Expression profiles of genes implicated in cell death/survival in the hippocampus of APP-Tg, Dhet, Npc1-null and ANPC compared to the wild-type mice

| **Gene Symbol** | **APP-Tg** | | **Dhet** | | **Npc1-null** | | **ANPC** | |
| --- | --- | --- | --- | --- | --- | --- | --- | --- |
|  | **Fold Regulation** | **p-value** | **Fold Regulation** | **p-value** | **Fold Regulation** | **p-value** | **Fold Regulation** | **p-value** |
| ***Akt1*** | -1.22 | 0.229 | -1.15 | 0.128 | -1.06 | 0.652 | -1.17 | 0.163 |
| ***Atg12*** | 1.07 | 0.582 | 1.07 | 0.591 | -1.02 | 0.824 | -1.08 | 0.559 |
| ***Atg5*** | -1.04 | 0.445 | -1.01 | 0.878 | -1.04 | 0.458 | -1.02 | 0.564 |
| ***Atg7*** | -1.17 | 0.454 | 1.00 | 0.966 | 1.06 | 0.652 | -1.26 | 0.117 |
| ***Bax*** | 1.02 | 0.784 | -1.04 | 0.432 | -1.05 | 0.391 | -1.15 | 0.077 |
| ***Bcl2*** | -1.03 | 0.760 | -1.19 | 0.265 | 1.01 | 0.946 | -1.16 | 0.315 |
| ***Becn1*** | 1.14 | 0.150 | 1.08 | 0.281 | -1.04 | 0.490 | -1.12 | 0.338 |
| ***Bid*** | -1.02 | 0.740 | -1.01 | 0.783 | 1.25 | 0.067 | 1.21 | 0.066 |
| ***Camk1*** | 1.19 | 0.313 | 1.03 | 0.908 | 1.10 | 0.593 | 1.04 | 0.818 |
| ***Capn5*** | 1.15 | 0.337 | -1.04 | 0.648 | -1.16 | 0.114 | -1.03 | 0.850 |
| ***Casp3*** | -1.06 | 0.483 | -1.10 | 0.256 | -1.15 | 0.054 | -1.20 | 0.056 |
| ***Cast*** | 1.22 | 0.174 | 1.06 | 0.682 | 1.30 | 0.149 | 1.14 | 0.319 |
| ***Cdk5*** | 1.14 | 0.340 | 1.00 | 0.999 | -1.26 | 0.08 | -1.29 | 0.097 |
| ***Ctsb*** | -1.09 | 0.199 | -1.26 | 0.006 | 1.13 | 0.111 | 1.25 | 0.011 |
| ***Ctsd*** | 1.14 | 0.382 | -1.04 | 0.681 | 2.20 | 0.000 | 2.38 | 0.000 |
| ***Fos*** | 1.85 | 0.472 | 1.05 | 0.940 | -1.43 | 0.378 | -1.11 | 0.571 |
| ***Gsk3b*** | 1.07 | 0.511 | -1.04 | 0.592 | -1.06 | 0.088 | -1.13 | 0.061 |
| ***Gusb*** | 1.11 | 0.252 | -1.08 | 0.424 | 1.53 | 0.002 | 1.54 | 0.001 |
| ***Igf2*** | -1.47 | 0.229 | -1.35 | 0.318 | 1.13 | 0.574 | -1.36 | 0.348 |
| ***Map1lc3b*** | 1.02 | 0.812 | 1.02 | 0.821 | -1.01 | 0.900 | -1.04 | 0.554 |
| ***Mapk1*** | -1.01 | 0.970 | 1.06 | 0.178 | 1.02 | 0.677 | 1.06 | 0.297 |
| ***Mapk3*** | -1.11 | 0.708 | -1.08 | 0.447 | -1.06 | 0.284 | -1.07 | 0.156 |
| ***Mapk8*** | -1.01 | 0.981 | 1.06 | 0.630 | -1.02 | 0.873 | -1.06 | 0.674 |
| ***Park2*** | 1.02 | 0.986 | -1.07 | 0.603 | 1.18 | 0.251 | 1.06 | 0.742 |
| ***Pik3c3*** | 1.13 | 0.315 | -1.08 | 0.323 | -1.15 | 0.029 | -1.05 | 0.658 |
| ***Pik3ca*** | -1.05 | 0.745 | -1.05 | 0.391 | -1.08 | 0.218 | -1.11 | 0.273 |
| ***Pin1*** | 1.12 | 0.075 | 1.00 | 0.961 | -1.03 | 0.615 | -1.04 | 0.407 |
| ***Pmaip1*** | 1.27 | 0.137 | 1.13 | 0.442 | 1.15 | 0.270 | 1.12 | 0.400 |
| ***Prdx2*** | 1.24 | 0.109 | -1.00 | 0.897 | -1.07 | 0.523 | -1.06 | 0.730 |
| ***Shisa5*** | 1.14 | 0.395 | 1.04 | 0.678 | 1.10 | 0.412 | 1.05 | 0.658 |

**Table S8.** Expression profiles of genes implicated in cell death/survival in cerebellum of APP-Tg, Dhet, Npc1-null and ANPC compared to the wild-type mice

| **Gene Symbol** | **APP-Tg** | | **Dhet** | | **Npc1-null** | | **ANPC** | |
| --- | --- | --- | --- | --- | --- | --- | --- | --- |
|  | **Fold Regulation** | **p-value** | **Fold Regulation** | **p-value** | **Fold Regulation** | **p-value** | **Fold Regulation** | **p-value** |
| ***Akt1*** | -1.03 | 0.731 | -1.06 | 0.488 | -1.11 | 0.249 | -1.01 | 0.926 |
| ***Atg12*** | 1.08 | 0.519 | 1.08 | 0.587 | -1.01 | 0.908 | 1.03 | 0.789 |
| ***Atg5*** | -1.03 | 0.735 | -1.04 | 0.777 | -1.11 | 0.353 | -1.08 | 0.189 |
| ***Atg7*** | -1.04 | 0.736 | -1.05 | 0.683 | -1.06 | 0.485 | 1.08 | 0.628 |
| ***Bax*** | 1.05 | 0.485 | -1.02 | 0.737 | -1.01 | 0.886 | -1.11 | 0.205 |
| ***Bcl2*** | 1.11 | 0.370 | 1.04 | 0.708 | -1.04 | 0.872 | 1.04 | 0.653 |
| ***Becn1*** | -1.07 | 0.533 | -1.11 | 0.309 | -1.14 | 0.189 | -1.06 | 0.648 |
| ***Bid*** | -1.01 | 0.870 | 1.09 | 0.546 | 2.25 | 0.007 | 2.56 | 0.001 |
| ***Camk1*** | -1.10 | 0.396 | -1.09 | 0.717 | 1.00 | 0.965 | -1.02 | 0.987 |
| ***Capn5*** | -1.17 | 0.024 | -1.15 | 0.312 | -1.12 | 0.228 | 1.06 | 0.624 |
| ***Casp3*** | -1.04 | 0.744 | -1.01 | 0.859 | 1.13 | 0.206 | -1.01 | 0.963 |
| ***Cast*** | 1.02 | 0.731 | 1.00 | 0.934 | 1.01 | 0.761 | -1.01 | 0.908 |
| ***Cdk5*** | -1.02 | 0.807 | 1.03 | 0.758 | -1.19 | 0.061 | -1.19 | 0.247 |
| ***Ctsb*** | -1.13 | 0.127 | -1.05 | 0.466 | 1.50 | 0.003 | 1.72 | 0.035 |
| ***Ctsd*** | -1.13 | 0.227 | -1.05 | 0.272 | 6.29 | 0.000 | 4.14 | 0.002 |
| ***Fos*** | -1.25 | 0.412 | 1.54 | 0.852 | 1.16 | 0.454 | 1.14 | 0.605 |
| ***Gsk3b*** | -1.08 | 0.091 | -1.11 | 0.259 | -1.09 | 0.052 | -1.05 | 0.380 |
| ***Gusb*** | -1.09 | 0.324 | -1.13 | 0.218 | 3.59 | 0.001 | 3.06 | 0.000 |
| ***Igf2*** | -1.11 | 0.884 | -1.01 | 0.951 | -1.07 | 0.633 | -1.29 | 0.342 |
| ***Map1lc3b*** | 1.04 | 0.701 | 1.11 | 0.390 | 1.13 | 0.304 | 1.20 | 0.158 |
| ***Mapk1*** | 1.06 | 0.183 | -1.01 | 0.808 | -1.07 | 0.163 | -1.06 | 0.356 |
| ***Mapk3*** | 1.08 | 0.455 | -1.03 | 0.775 | 1.00 | 0.972 | 1.04 | 0.680 |
| ***Mapk8*** | 1.00 | 0.992 | 1.06 | 0.732 | 1.05 | 0.741 | -1.00 | 0.972 |
| ***Park2*** | -1.22 | 0.032 | -1.18 | 0.068 | 1.28 | 0.005 | 1.19 | 0.267 |
| ***Pik3c3*** | 1.07 | 0.194 | -1.09 | 0.219 | -1.19 | 0.049 | -1.17 | 0.241 |
| ***Pik3ca*** | -1.32 | 0.306 | -1.11 | 0.360 | -1.15 | 0.044 | -1.19 | 0.022 |
| ***Pin1*** | 1.03 | 0.697 | -1.04 | 0.543 | -1.06 | 0.459 | -1.02 | 0.840 |
| ***Pmaip1*** | 1.02 | 0.927 | 1.10 | 0.501 | 1.43 | 0.036 | 1.31 | 0.041 |
| ***Prdx2*** | -1.11 | 0.530 | -1.09 | 0.213 | -1.14 | 0.091 | -1.21 | 0.313 |
| ***Shisa5*** | -1.07 | 0.612 | 1.02 | 0.883 | 1.36 | 0.039 | 1.23 | 0.262 |
